# Association between behavioral addiction and psychological disorders among medical students in Egypt, Sudan, and Libya: a cross-sectional study

**DOI:** 10.1038/s41598-026-56057-9

**Published:** 2026-06-04

**Authors:** Abdelmonam M. Hagag, Abdalrahman Salah Shehata, Athar Mohamed Abdelhamed, Gsemallah Abdo Ahmed, Omar A. Ghanem, Ayoub Akwaisah, Mohamed Abdelghani

**Affiliations:** 1 Faculty of Medicine, Zagazig University, Zagazig, Egypt; 2 Faculty of Medicine, Qena University, Qena, Egypt; 3 Faculty of Medicine, Merowe University of Technology, Merowe, Sudan; 4 Faculty of Medicine, Benghazi University, Benghazi, Libya; 5 Psychiatry and Addiction Department, Zagazig University, Zagazig, Egypt

**Keywords:** Behavioral addiction, Problematic internet use, Gaming disorder, Pornography addiction, Psychological disorders, Stress, Anxiety, Depression, Cross-sectional study, Diseases, Health care, Psychology, Psychology, Risk factors

## Abstract

We aimed to assess the prevalence of problematic internet use, gaming disorder symptoms, or problematic pornography consumption among medical students in Egypt, Sudan, and Libya, and their associations with psychological disturbances. A cross-sectional study was conducted via an online questionnaire. We used the Problematic Internet Use Scale (IAT), the Internet Gaming Disorder Scale–Short-Form (IGDS9-SF), and the Problematic Pornography Consumption Scale (PPCS). Chi-square tests were used to assess the variation between participants on the basis of their nationality. Multivariable multiple linear regression was used to assess the associations between behavioral addiction and psychological disorders and other confounders. A total of 1284 patients completed the questionnaire. The prevalence rates were 28.82%, 6.78%, and 7.55% for problematic internet use, gaming disorder symptoms, or problematic pornography consumption, respectively. Compared with other students, Egyptian medical students had a significant increase in all behavioral addictions. A positive association between increased behavioral addictions and anxiety or depression was observed. Stress was positively associated with problematic internet use and negatively associated with gaming disorder and pornography addiction. Sleeping less than 6 h was negatively associated with gaming disorder symptoms or problematic pornography consumption, whereas sleeping more than 9 h was positively associated with gaming disorder symptoms or problematic pornography consumption. Our study revealed a moderate level of problematic internet use, with a low level of gaming disorders and pornography addiction, alongside significant regional variation in prevalence.

**Supplementary Information:**

The online version contains supplementary material available at 10.1038/s41598-026-56057-9.

## Introduction

In this period of time, almost everything in our world changed to data; we even call it the digital world, where everything people want to do they have done it by using the internet. The internet has become a major tool for performing essential tasks in one’s life, such as communication, shopping, education, gaming, and entertainment^1^. With respect to the rare use of social media, which involves applications and online channels, such as Facebook, YouTube, WeChat, and QQ, these programs help us interact with others, share useful and interesting content, and alleviate psychological distress, especially among students who experience stress resulting from pressures such as academic competitions, employment prospects, and self-identity^2^.

Despite its importance, unfortunately, excessive use of the internet can lead to addiction^3^. Goldberg was the first person to use the expression “Problematic internet use” as a prediction in 1995^4^. Problematic internet use refers to the excessive use of the internet for at least 6 months, with five or more of the following criteria: preoccupation; spending more time to achieve more gratification; unsuccessful attempts to reduce the usage of the internet; experiencing a negative mood when not using it; a negative impact on academic, social, or work performance; and using the internet to cope with problems^5^.

Additionally, gaming disorder is another behavioral addiction that affects adolescents. It is defined as the existence of a dysfunctional gaming disorder that has five or more of the following characteristics: preoccupation, psychological withdrawal, tolerance, failure to cut off its use, loss of interest in other habits, continuing game despite problems, deceiving family members or others about the time of using it, using gaming to cope with problems, and risk of losing jobs or relationships due to gaming^6^. Finally, excess use of the internet was associated with a significant increase in Problematic pornography use, which manifests in several forms, such as sexual arousal, via images, videos, or written materials^7^.

For college students, especially medical students, the internet has become an essential tool and part of their everyday life. They use it for education (online lectures and conferences), research, medical applications, and other online resources^8^. Several studies have shown that college students have a high prevalence and a high incidence of problematic internet use^9^. A previous study conducted among 26 countries worldwide reported a prevalence of approximately 38.5%^10^, whereas another cross-sectional study among medical students in Sudan reported that the prevalence of problematic internet use was 75.5%, of which 39.7% were mild, 33.2% were moderate, and 2.6% were severe^11^. On the other hand, the prevalence of gaming disorders is lower than that of problematic internet use, which ranges from 0.9% to 19.9% according to a previous meta-analysis, whereas Pitanupong et al. 2025 reported a prevalence of gaming disorder of 8.4% among medical students in Thailand^12^.

Finally, the prevalence of problematic pornography use ranged from 3.2% to 16.6% worldwide, with a negative association between it and education level. In a recent cross-sectional study among nurse students in Egypt, Karim et al.^13^ reported a significant association between internet use and problematic pornography use. This led the WHO to classify this phenomenon as a cumulative sexual behavioral disorder^14^. All three types of behavioral addictions significantly increase the incidence of psychological disorders, which has been reported in previous cross-sectional studies^12,13,15,16^.

In the Middle East, few studies have attempted to assess the prevalence of problematic internet use and its association with increased depression, anxiety, and stress among medical students^16,17^. However, studies on gaming disorder and problematic pornography use are lacking. In this study, we aim to estimate the prevalence of these three addictive behaviors (Internet, gaming, and problematic pornography use) in three Arab countries (Egypt, Sudan, and Libya). We also aimed to assess the relationships among these three addictive behaviors and their relationships with increased stress, anxiety, and depression in this population. In the end, all these objectives lead to improved awareness of the internet, gaming, and problematic pornography use among medical students in the MENA region to help improve their quality of life. We hypothesize a high prevalence of internet and gaming disorder, with low problematic pornography use among this population. We also hypothesize that there is a significant association between these behavioral addictions and psychological disorders. Finally, we chose these three countries because of their geographical proximity and shared Arabic culture.

## Methodology

### Study design and setting

We conducted a cross-sectional study among medical students and interns in three Middle East and North African countries: Egypt, Sudan, and Libya. Data were collected from August 2025 to December 2025. We followed the STROBE guidelines^18^ in reporting cross-sectional studies. Ethical approval was obtained from each county involved in the study. Approval was obtained from Zagazig University, Faculty of Medicine (ZU-IRB # 1547/29-July 2025), University of Merowe for Technology in Sudan (MUT 001), and Benghazi Medical Center from Libya (NBC: 005. H. 25. 10).

### Sample size

The sample size was calculated via the Epi Info calculator^19^. We expected the prevalence of problematic internet use to be 15.1%^16^, with 95% confidence intervals and a 5% margin of error. The required sample size was 197 for each country. We also added a 10% nonresponse rate, so the final sample size was 216 for each country, revealing a total sample size of 684.

### Study population

The study population included medical students who were from the faculty of medicine only in the selected countries, who had access to online platforms and agreed to participate in the study. They were excluded if they refused to participate. Participants who were paramedical, pharmacy, or denial students were excluded.

### Data collection method

Data were collected through an online questionnaire distributed via social media platforms, including WhatsApp, Facebook, and Telegram. We preferred the online version of the questionnaire because it was easy to reach the wide range of the target population to help us collect the target sample size with ease. One of the authors of the study designed the questionnaire via Google Forms, and it underwent a technical check by the first author to ensure the accuracy of the questionnaire. The questionnaire was prepared in English via validated versions of the included scales. Finally, to ensure that each participant would participate only once, we allowed for only one response in the Google form setting to allow the participant to fill out the form only once, and the average time for completion was approximately 10 min.

The survey consisted of eight sections, and the participants were not allowed to omit any section. In the first section, we identified the importance of the study, explained the purpose of the study, and ensured that the participants’ responses were confidential and would be used only for research purposes. We also obtained informed consent from the participants. No other promotion was provided to the participants, and no fees were paid to the participants. The second section collected sociodemographic data such as age, sex, type of university, smoking level, number of sleeping hours, difficulty accessing the internet, and reason for using internet platforms. All the sociodemographic data, including sleep duration, from the participants were subjective information assessed via multiple-choice questions, and no validated questionnaire was used to assess any of the data, including sleep hours. For sleeping hours, the question was also a multiple-answer question containing three answers: less than 6 h, 7–8 h, and more than 9 h, and the students were asked to choose one answer on the basis of their usual sleep duration.

The third section measures problematic internet use via the problematic internet use Scale (IAT)^20,21^, which is a 20-item Likert scale, and each question has six answer scales ranging from 0 to 5 (not applicable, rarely, occasionally, frequently, often, and always), with total scores ranging from 0 to 100. The participants were classified into normal usage, mild, moderate, and severe levels of addiction if they scored (0–30), (31–49), (50–79), and (80–100), respectively. We also used a score of ≥ 50 as a cutoff value to indicate the presence of problematic internet use^22^. The scale has excellent internal consistency (α: 0.9–0.93) and good test–retest reliability (r: 0.85)^23^.

Gaming disorder was assessed in the fourth section via the internet Gaming Disorder Scale–Short-Form (IGDS9-SF)^24^. This scale consists of a 9-point Likert scale. Each question has five answers, which are scored from 1 to 5. The total score ranges from 9 to 45, with a higher score indicating a higher level of gaming disorder, whereas a score of ≥ 32 indicates the presence of gaming disorder^25^. The scale showed excellent internal consistency (α: 0.87) and good test-retest reliability (r: 0.86).

The fifth section evaluated anxiety levels via the Generalized Anxiety Disorder-7 (GAD-7)^26^ scale, which consists of a 7-point Likert scale, with four answers scored from 0 to 3 and scores ranging from 0 to 21. Anxiety severity was categorized as minimal, mild, moderate, or severe if the score was (0–4), (5–9), (10–14), or (15–21), respectively. We also used a score of 10 or more as a cutoff value to indicate the presence of anxiety^26^. Additionally, it showed excellent internal consistency (α 0.89–0.92) and good test–retest reliability (*r* = 0.83).

In the sixth section, we used the Patient Health Questionnaire-9 (PHQ-9)^27^ to assess the level of depression. A 9-point Likert scale with four answers for each question was used. Scores range from 0 to 3, with scores ranging from 0 to 27. Students were classified into minimal, mild, moderate, moderately severe, and severe levels of depression if they scored (0–4), (5–9), (10–14), (15–19), and (20–27), respectively, and a score of 10 or more was used to detect the presence of depression^28^. The scale again showed excellent internal consistency (α = 0.89) and good test‒retest reliability (*r* = 0.84).

Stress levels were evaluated in the seventh section through the Perceived Stress Scale-10 (PSS-10)^29^. A 10-point Likert scale is used, and each question has 5 answers, which are scored from 0 to 4. The total score ranges from 0 to 40. The students were classified into mild, moderate, or severe stress groups if they scored 0–13, 14–26, or 27–40, respectively, and a score of 27 or more was considered the stress level. It showed good internal consistency (α: >0.7) and good test-retest reliability (r: >0.7).

Finally, the eighth section assessed problematic pornography consumption via the Problematic Pornography Consumption Scale (PPCS)^30^, which consists of an 18-item Likert scale. Each question consists of 7 answers, and their scores range from 1 to 7. The total score ranges from 18 to 126, with higher scores indicating greater severity; a cutoff score of 76 was applied to identify individuals at high risk. The psychometric validation revealed strong internal consistency (α: 0.93).

### Pilot study

A pilot study was first conducted in each country to assess the reliability and readiness of the questionnaire before the main study started. We selected 5–10% of the required samples, with approximately 20 in Egypt, 20 in Sudan, and 10 in Libya. We used a convenience sampling technique to collect the intended population in the pilot study; for every responder, we asked about any difficulty in the readiness of the questionnaire, and if one of them was reported, it was assessed and modified. We also assessed reliability via McDonald’s Omega (Ω), which resulted in strong consistency and reliability of the results (the results ranged from 0.91 for the PHQ-9 to 0.98 for the PPCS). See Table S1 in the supplementary materials. The participants in the pilot study were excluded from the results.

### Statistical methods

The Google form questionnaire required a mandatory response for all items; therefore, item-based level missing data were negligible in the final analytical database. Consequently, the analysis was performed based on complete-case analysis, and no imputation procedures were required. Given the force-response design, the formal assessment of missingness mechanisms (MCAR/MAR/MNAR) was not considered necessary.

Continuous variables are summarized using medians and interquartile ranges (IQRs), and categorical variables are summarized using frequencies and percentages and were compared via the Pearson chi-square test. Spearman’s correlation was used to test the correlation between the different scales. Additionally, internal reliability for each scale was assessed via McDonald’s Omega (Ω); omega values between > 0.65 and 0.80 were considered acceptable, values > 0.80 were considered strong, and values below 0.65 were considered unacceptable^31^.

Prevalence was compared across subgroups via Pearson’s chi-square test. Prevalence reported with the corresponding 95% confidence interval (CI) calculated via the Wilson method. A multivariate multiple linear regression model was used to assess variables associated with behavioral addiction across the three correlated scales (IAT, PPCS, and IGDS). This approach accounts for the covariation between the scales, reducing the risk of inflated Type I error that could arise from separate univariate models. Furthermore, MANOVA with Pillai’s trace was used to calculate the omnibus p value for overall factor significance. We used the generalized variance inflation factor (GVIF) and adjusted GVIF (aGVIF) to assess collinearity among the predictors.

To explore whether the relationships between stress or sleep duration and behavioral addiction were mediated by psychological distress, we fitted four parallel dual-mediator structural equation models. Depression (PHQ-9 score) and anxiety (GAD-7 score) were specified as parallel mediators in all the models, with their residual variance freely estimated.

In the first two models, perceived stress (PSS score) was specified as exposure, and problematic internet use (IAT score) or pornography addiction (PPCS score) served as the outcome. In the mediator equation, the PHQ-9 and GAD-7 scores were regressed on the PSS score and adjusted for age, sex and study country. In the outcome equation, the addiction score was regressed on the PSS score, both of which are mediators, and adjusted for age, sex, study country, income and sleep duration.

In the second pair of models, sleep duration was the exposure, with the IAT score or PPCS score as the outcome. Sleep was entered simultaneously as two dummy variables (< 6 h and > 9 h vs. the 7–8-h reference) in both the mediator and outcome equations. The PHQ-9 and GAD-7 scores were regressed on both sleep dummies. The PHQ-9 and GAD-7 scores were regressed on both sleep dummies, with PSS score, age, sex, and country as covariates. The outcome was regressed on both mediators and both sleep dummies, with PSS score, age, gender, country, and income as additional covariates.

For the PSS models, three indirect effects were estimated per model: the pathway through depression (a1×b1), the pathway through anxiety (a2×b2), and their sum as the total indirect effect. For the sleep models, six indirect effects were estimated per model: one pair of mediator pathways for each sleep dummy variable (a1_less×b1, a2_less×b2, and their sum; a1_more×b1, a2_more×b2, and their sum), two direct effects (c′_less and c′_more) and two total effects. All standard errors (SEs) and CIs were derived from 5000 bootstrap resamplings via the percentile method. Model fit was evaluated via the chi-square test, degrees of freedom, chi-square/df ratio, Bollen–Stine bootstrap p value, comparative fit index (CFI), Tucker‒Lewis index (TLI), standardized root mean square residual (SRMR), and root mean square error of approximation (RMSEA) with 90% CIs.

Furthermore, to identify empirically derived subgroups with distinct patterns of behavioral addiction and psychological distress, we conducted latent profile analysis (LPA). LPA was conducted on six continuous scales (IAT, PPCS, IGDS, PHQ-9, GAD-7, PSS). Equal-variance, varying-means models were estimated across 1–15 latent classes. Model selection was guided by multiple fit indices, including the Akaike information criterion (AIC), Bayesian information criterion (BIC), sample-size-adjusted BIC (SABIC), and bootstrap likelihood ratio test (BLRT). The classification quality was assessed via entropy and the minimum posterior class membership probability (prob_min). The 5-class solution was selected on the basis of acceptable entropy (0.79), adequate minimum class membership probability (0.66), interpretability of the resulting profiles, and avoidance of near-empty classes observed in solutions with six or more classes (supplementary material). The P value was two-sided, with *p* < 0.05 considered significant. All analyses were conducted via R software (version 4.4.2, version 4.4.2; R Core Team 2024). The packages used included tidyverse, ggplot2, lavaan (v0.6-21), tidyLPA, and gtsummary^32–34^. A fixed random seed (123) was used throughout the analysis to ensure the reproducibility of the stochastic procedures.

## Results

### Sociodemographic data

**Fig. 1:**
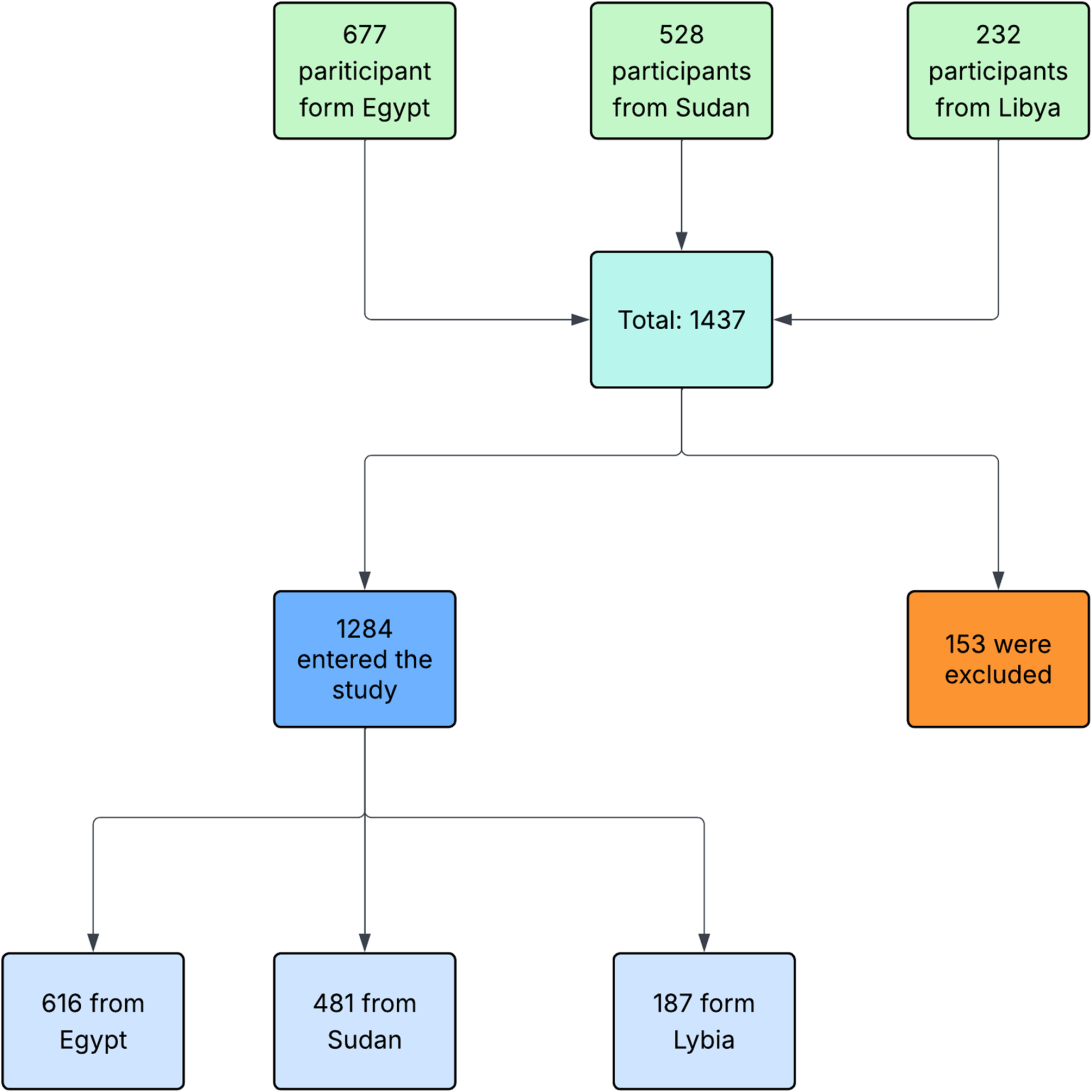
Flow chart of the distribution of the participants in the three countries.

During the data collection period, 1,437 participants completed the questionnaire (677 from Egypt, 528 from Sudan, and 232 from Libya). Among the participants, 153 (10.65%) were excluded because they were not eligible. The reasons for exclusion were nonmedical students or refusal to participate. The remaining 1284 eligible responses were 616 from Egypt, 481 from Sudan, and 187 from Libya. See Table 1; Fig. 1.

**Table 1:** Baseline characteristics of the included participants.

Characteristic	Overall	Egypt	Libya	Sudan
	*N* = 1,284^1^	*N* = 616^1^	*N* = 187^1^	*N* = 481^1^
Age (year)	23 (21, 24)	22 (20, 23)	25 (24, 27)	23 (21, 25)
Gender				
Female	795 (62%)	379 (62%)	138 (74%)	278 (58%)
Male	489 (38%)	237 (38%)	49 (26%)	203 (42%)
University type				
National	79 (6.2%)	51 (8.3%)	6 (3.2%)	22 (4.6%)
Private	185 (14%)	27 (4.4%)	15 (8.0%)	143 (30%)
Public	1,020 (79%)	538 (87%)	166 (89%)	316 (66%)
Study year				
First	40 (3.1%)	24 (3.9%)	10 (5.3%)	6 (1.2%)
Second	236 (18%)	101 (16%)	8 (4.3%)	127 (26%)
Third	146 (11%)	72 (12%)	16 (8.6%)	58 (12%)
Forth	278 (22%)	122 (20%)	33 (18%)	123 (26%)
Fifth	337 (26%)	174 (28%)	59 (32%)	104 (22%)
Six	25 (1.9%)	0 (0%)	9 (4.8%)	16 (3.3%)
Intern	222 (17%)	123 (20%)	52 (28%)	47 (9.8%)
Study phase				
Academic phase	480 (37%)	189 (31%)	46 (25%)	245 (51%)
Clinical phase	590 (46%)	305 (50%)	87 (47%)	198 (41%)
Intern	214 (17%)	122 (20%)	54 (29%)	38 (7.9%)
Sleep hours				
Less than 6 h	398 (31%)	206 (33%)	63 (34%)	129 (27%)
7–8 h	827 (64%)	376 (61%)	120 (64%)	331 (69%)
More than 9 h	59 (4.6%)	34 (5.5%)	4 (2.1%)	21 (4.4%)
Income				
Enough and saving	555 (43%)	273 (44%)	90 (48%)	192 (40%)
Enough, but not saving	620 (48%)	305 (50%)	86 (46%)	229 (48%)
Not enough	109 (8.5%)	38 (6.2%)	11 (5.9%)	60 (12%)
Residency				
Rural	480 (37%)	266 (43%)	33 (18%)	181 (38%)
Urban	804 (63%)	350 (57%)	154 (82%)	300 (62%)
Living place				
Living alone	61 (4.8%)	39 (6.3%)	5 (2.7%)	17 (3.5%)
Living with family	922 (72%)	475 (77%)	180 (96%)	267 (56%)
Living with friends	301 (23%)	102 (17%)	2 (1.1%)	197 (41%)
Smoking				
No	1,226 (95%)	599 (97%)	174 (93%)	453 (94%)
Yes	58 (4.5%)	17 (2.8%)	13 (7.0%)	28 (5.8%)
Family history of mental disease				
No	1,120 (87%)	535 (87%)	164 (88%)	421 (88%)
Yes	164 (13%)	81 (13%)	23 (12%)	60 (12%)
Internet connection difficulty				
No	1,114 (87%)	575 (93%)	162 (87%)	377 (78%)
Yes	170 (13%)	41 (6.7%)	25 (13%)	104 (22%)
Marital status				
Single	1,215 (95%)	602 (98%)	168 (90%)	445 (93%)
Married	63 (4.9%)	14 (2.3%)	16 (8.6%)	33 (6.9%)
Previously married	6 (0.5%)	0 (0%)	3 (1.6%)	3 (0.6%)
Chronic disease				
No	1,153 (90%)	555 (90%)	169 (90%)	429 (89%)
Yes	131 (10%)	61 (9.9%)	18 (9.6%)	52 (11%)
Academic grade				
Excellent	459 (36%)	334 (54%)	46 (25%)	79 (16%)
Very good	424 (33%)	179 (29%)	67 (36%)	178 (37%)
Good	350 (27%)	79 (13%)	60 (32%)	211 (44%)
Fair	43 (3.3%)	21 (3.4%)	12 (6.4%)	10 (2.1%)
Failed	8 (0.6%)	3 (0.5%)	2 (1.1%)	3 (0.6%)
Grade satisfaction				
Very unsatisfied	49 (3.8%)	25 (4.1%)	3 (1.6%)	21 (4.4%)
Unsatisfied	208 (16%)	112 (18%)	21 (11%)	75 (16%)
Natural	405 (32%)	183 (30%)	66 (35%)	156 (32%)
Satisfied.	466 (36%)	228 (37%)	67 (36%)	171 (36%)
Very satisfied	156 (12%)	68 (11%)	30 (16%)	58 (12%)
Life satisfaction				
Very unsatisfied	29 (2.3%)	20 (3.2%)	2 (1.1%)	7 (1.5%)
Unsatisfied	134 (10%)	82 (13%)	18 (9.6%)	34 (7.1%)
Natural	438 (34%)	221 (36%)	65 (35%)	152 (32%)
Satisfied	449 (35%)	216 (35%)	71 (38%)	162 (34%)
Very satisfied.	234 (18%)	77 (13%)	31 (17%)	126 (26%)

^1^Median (Q1, Q3); n (%).

The median age of the participants was 23 (21–24), 62% were females, and 79% were from public universities. Most students were in their fourth and fifth years (22% and 26%, respectively), and 17% were medical interns. Approximately 64% of the participants slept for approximately 7–8 h. Only 13% had internet connection difficulty. Finally, approximately half of them were satisfied with their academic performance, and 53% were satisfied with their life. See Table 1.

### Prevalence

**Fig. 2:**
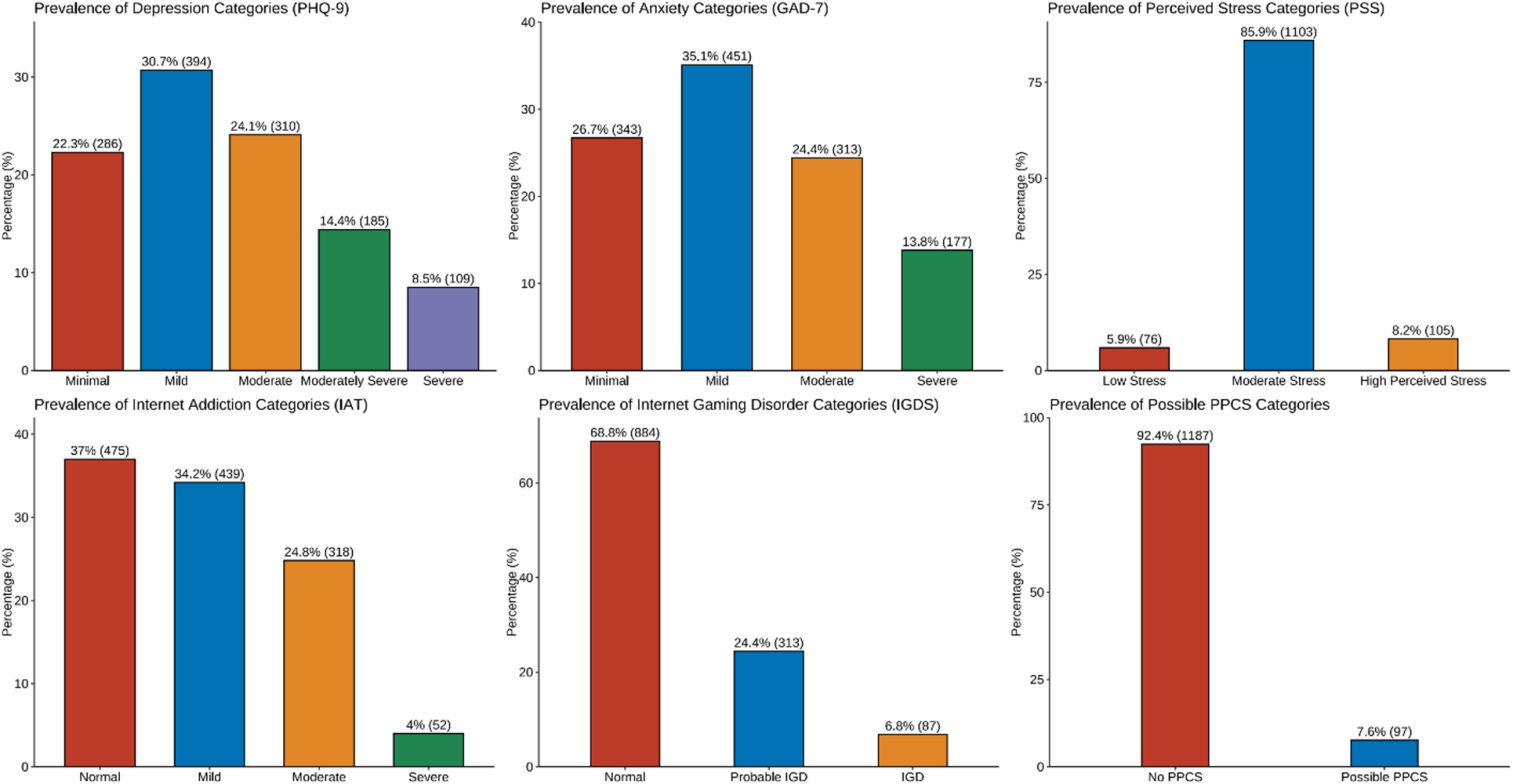
Prevalence of each category at each scale.

The prevalence of problematic internet use varies among countries. The overall prevalence was approximately 28.82% (95% CI (26.41%–31.35%)). Egypt had the highest prevalence, at approximately 35%, followed by Sudan, at 27%, and Libya, at only 16% (P value: <0.001). The same finding was observed for both gaming disorder and problematic pornography use. The overall prevalence of gaming disorder was 6.78% (95% CI: 5.53%–8.28%), whereas in Egypt, the prevalence was 9.3%; in Sudan, it was 5.2%; and in Libya, it was 2.7% (P value: 0.002). For Problematic pornography use, the overall prevalence was approximately 7.55% (95% CI: (6.23%–9.13%)), whereas in Egypt, it was 9.3%; in Sudan, it was 6.7%; and in Libya, it was 4.3% (P value: <0.001). See Table 2; Fig. 2, and Table S2 in the supplementary material.

**Table 2:** Absolute prevalence of the selected variables.

Variable	n (*N* = 1284)	Prevalence (95% CI)
Problematic internet use (IAT) (cutoff ≥ 50)	370	28.82% (26.41%–31.35%)
Porn addiction (PPCS) (cutoff ≥ 76)	97	7.55% (6.23%–9.13%)
Gaming disorder (IGDS) (cutoff ≥ 32)	87	6.78% (5.53%–8.28%)
Depression prevalence (PHQ-9) (cutoff ≥ 10)	604	47.04% (44.32%–49.78%)
Anxiety (GAD-7) (cutoff ≥ 10)	490	38.16% (35.54%–40.85%)
Stress (PSS) (cutoff ≥ 27)	105	8.18% (6.8%–9.8%)

CI, confidence interval calculated using the Wilson method.

### Comparison of participants’ prevalence according to their baseline characteristics

The chi-square results revealed a statistically significant difference in the prevalence of both gaming disorder and problematic pornography use between males and females, as males presented a greater prevalence of both addictions (12% vs. 5%) and (8.6% vs. 5.7%) (P values: <0.001 and 0.043) for both problematic pornography use and gaming disorders, respectively. See Table S3 in the supplementary materials. We also found a significant positive association with problematic internet use among rural residents compared with urban residents (32% vs. 27%, P value: 0.034). See Table S7 in the supplementary materials.

Our univariate analysis revealed that smokers had a higher prevalence of problematic pornography use than nonsmokers did (17% vs. 7.1%, P value: 0.009). See Table S9 in the supplementary materials. Additionally, the highest prevalence of both problematic internet and problematic pornography use was found in the participants who were very unsatisfied and unsatisfied with their academic grade, whereas the prevalence declined in the satisfied and very satisfied participants. The same findings were observed for the participants’ satisfaction with their academic life. See Tables S10–S15 in the supplementary materials.

### Correlation and regression

**Fig. 3:**
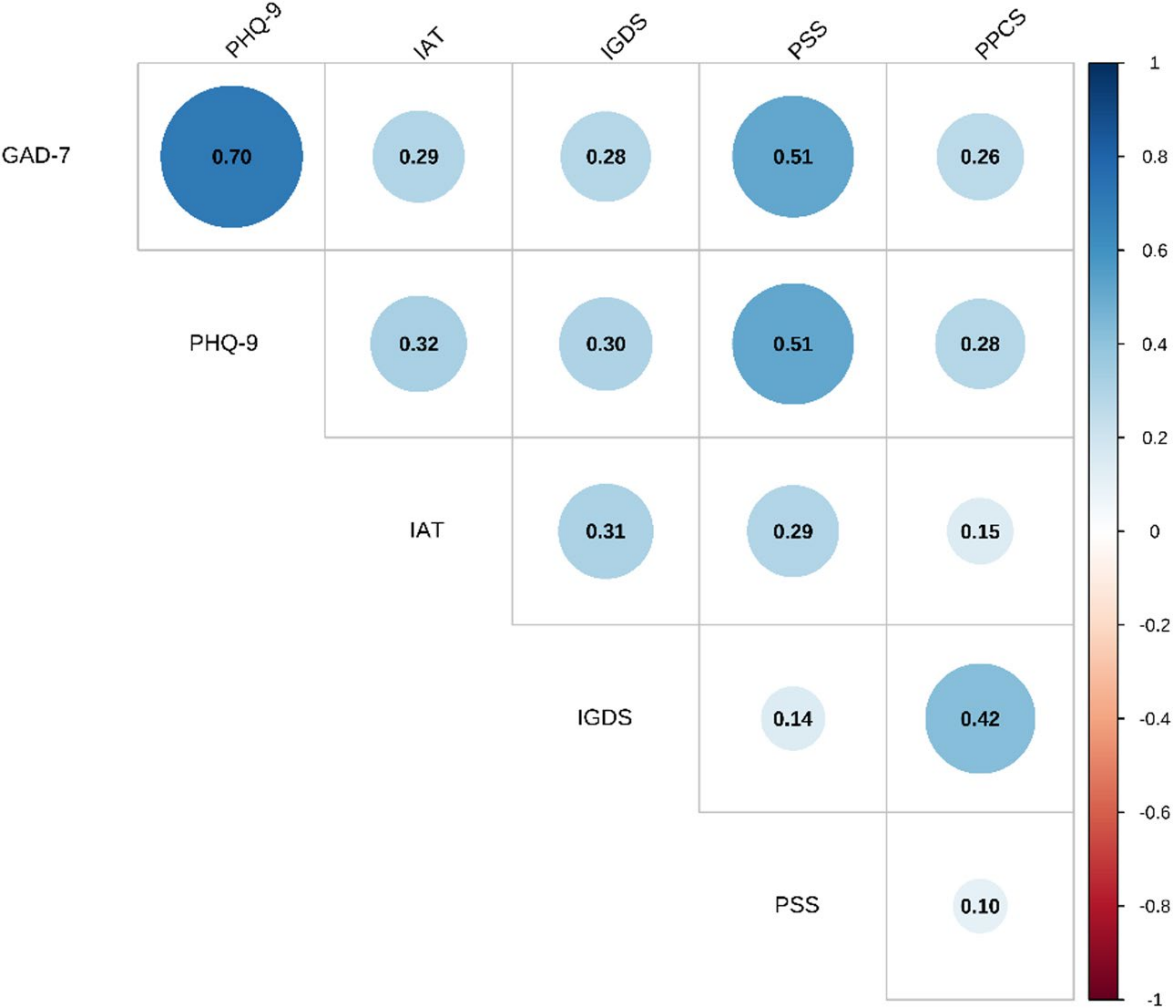
Correlation plot between the six different scales.

Spearman’s correlation between the continuous data revealed a positive correlation between the 6 continuous variables. As shown in Fig. 3, the highest correlation was observed for the PH-9 and GAD-7 scales (0.7). A good positive correlation was also observed between problematic internet use and both gaming and problematic pornography use (Spearman rho: 0.31 and 0.15, respectively). See Fig. 2.

Colinearity assessment models for problematic internet use, gaming disorder, and problematic pornography use. There was no evidence of multicollinearity between the predictors, as all the adjusted generalized variance inflation factors (aGVIFs) were less than 2.3. These results support the assumption of the multivariable linear regression model. See Tables S16 and S17 in the supplementary materials.

Multivariate multiple linear regression was performed to assess the associations between internet, gaming, and problematic pornography use and other variables, including stress, anxiety, and depression levels. We found associations between psychological disorders and problematic internet use, gaming disorder, and problematic pornography use. An increase in the anxiety score was positively associated with the problematic internet use score of approximately 0.41, the gaming disorder score of approximately 0.22, and the problematic pornography use score of approximately 0.53. (β: 0.41, 95% CI 0.15, 0.67), P value: 0.002), (β: 0.22, 95% CI 0.11, 0.33), P value: <0.001), and (β: 0.53, 95% CI 0.23, 0.83), P value: 0.001), for problematic internet use, gaming disorder, and problematic pornography use, respectively. Additionally, an increase in the depression score was positively associated with the problematic internet use score of 0.52, the gaming disorder score of 0.26, and the problematic pornography use score of 0.85. (β: 0.52, 95% CI 0.3, 0.74), P value: <0.001), (β: 0.26, 95% CI 0.17, 0.35), P value: <0.001), (β: 0.8, 95% CI 0.54, 1.05), P value: <0.001), for internet, gaming, and problematic pornography use, respectively.

Stress was positively associated with the problematic internet use score of 0.45, whereas it was negatively associated with gaming disorder by approximately − 0.17 and a negative association with the problematic pornography use score by approximately − 0.54. (β: 0.45, 95% CI 0.16, 0.74), P value: 0.003), (β: − 0.17, 95% CI − 0.29, − 0.05), P value: 0.006), (β: − 0.54, 95% CI − 0.87, − 0.2), P value: 0.002), for internet, gaming, and problematic pornography use, respectively. See Table 3.

**Table 3:** Multivariate multiple linear regression.

Variable	IAT score		IGDS score		PPCS score		MANOVA *p* value^1^
	B (95% CI)	*p* value	B (95% CI)	*p* value	B (95% CI)	*p* value	
Age	− 0.29 (− 0.76, 0.18)	0.228	0.05 (− 0.14, 0.24)	0.6	− 0.35 (− 0.89, 0.19)	0.203	0.244
Gender							0.285
Female	Ref.	–	–	–	–	–	–
Male	1.53 (− 7.15, 10.21)	0.73	− 2.01 (− 5.58, 1.55)	0.269	− 8.23 (− 18.17, 1.72)	0.105	–
GAD-7 score	0.41 (0.15, 0.67)	**0.002**	0.22 (0.11, 0.33)	**< 0.001**	0.53 (0.23, 0.83)	**0.001**	**< 0.001**
PHQ-9 score	0.52 (0.3, 0.74)	**< 0.001**	0.26 (0.17, 0.35)	**< 0.001**	0.8 (0.54, 1.05)	**< 0.001**	**< 0.001**
PSS score	0.45 (0.16, 0.74)	**0.003**	− 0.17 (− 0.29, − 0.05)	**0.006**	− 0.54 (− 0.87, − 0.2)	**0.002**	**< 0.001**
Study country							**0.002**
Egypt	Ref.	–	–	–	–	–	–
Libya	− 5.79 (− 9.14, − 2.44)	**0.001**	− 2.23 (− 3.61, − 0.85)	**0.002**	0.04 (− 3.8, 3.88)	0.984	–
Sudan	− 2.6 (− 4.92, − 0.29)	**0.027**	− 0.87 (− 1.82, 0.08)	0.073	1.39 (− 1.26, 4.04)	0.304	–
Income							0.297
Enough and saving	Ref.	–	–	–	–	–	
Enough, but not saving	− 0.77 (− 2.86, 1.33)	0.473	0.62 (− 0.24, 1.48)	0.158	− 0.21 (− 2.61, 2.2)	0.867	
Not enough	− 3.81 (− 7.62, 0)	0.05	0.02 (− 1.54, 1.59)	0.976	0.34 (− 4.03, 4.71)	0.878	
Sleep hours							**0.009**
7 to 8 h	Ref.	–	–	–	–	–	–
Less than 6 h	− 2.74 (− 4.94, − 0.55)	**0.014**	− 0.77 (− 1.67, 0.13)	0.094	− 0.87 (− 3.39, 1.64)	0.496	–
More than 9 h	0.99 (− 3.83, 5.82)	0.687	2.21 (0.23, 4.19)	**0.029**	7.11 (1.58, 12.64)	**0.012**	–
PSS score × gender [Male] interaction	− 0.06 (− 0.49, 0.37)	0.787	0.24 (0.06, 0.42)	**0.009**	0.96 (0.46, 1.46)	**< 0.001**	**< 0.001**

CI, confidence interval; Ref., reference level; IAT, problematic internet use Test; IGDS, internet Gaming Disorder Scale; PPCS, Problematic Pornography Consumption Scale; GAD-7, Generalized Anxiety Disorder-7; PHQ-9, Patient Health Questionnaire-9; PSS, Perceived Stress Scale.

^1^Pillai’s trace for overall factor significance.

Compared with being in Egypt, being in Libya or Sudan was significantly negatively associated with the problematic internet use score, whereas there was no difference in the problematic pornography use score. For gaming disorder, a significant negative association was observed only with the Libyan results, whereas a numerical reduction in the gaming score was observed in the Sudanese results. See Table 3. On the other hand, we did not find any association between behavioral addiction and gender. See Table 3.

Finally, sleeping less than 6 h was significantly negatively associated with problematic internet use, whereas sleeping more than 9 h was positively associated with problematic internet use, but the difference was not statistically significant. The same findings were observed for both gaming disorder and problematic pornography use, as sleeping less than 6 h was negatively associated with gaming disorder and problematic pornography use, whereas sleeping more than 9 h was positively associated with gaming disorder and problematic pornography use. See Table 3.

The structural equation model (SEM) showed excellent model fit for the variables assessed. With respect to the perceived stress models, both the internet and problematic pornography uses showed excellent fit indices (X^2^/df: 2.15, P value: 0.028, Bollen-Stine p: > 0.99, CFI: 0.994, TLI: 0.978, SRMR: 0.014, RMSEA: 0.03, 90% CI (0.009–0.05) for the problematic internet use model and (X^2^/df: 2.15, P value: 0.028, Bollen-Stine p: >0.99, CFI: 0.994, TLI: 0.978, SRMR: 0.015, RMSEA: 0.03, 90% CI (0.009–0.05) for the problematic pornography use model. See Table S18. The same was found for sleep, as it also showed excellent model fit for both internet and problematic pornography use. (X^2^/df: 3.02, P value: 0.017, Bollen-Stine p: >0.99, CFI: 0.995, TLI: 0.962, SRMR: 0.012, RMSEA: 0.04, 90% CI (0.015–0.066) for problematic internet use and (X^2^/df: 3.02, P value: 0.017, Bollen-Stine p: >0.99, CFI: 0.995, TLI: 0.962, SRMR: 0.013, RMSEA: 0.04, 90% CI (0.015–0.66) for problematic pornography use. See Figs. S18–S22 in the supplementary materials.

Perceived stress was associated with greater depression (β: 0.69, 95% CI (0.63 to 0.76), P value: <0.001) and anxiety levels (β: 0.58, 95% CI (0.52 to 0.63), P value: <0.001). In addition, both anxiety and depression were positively associated with problematic internet use and problematic pornography use. In the problematic internet use model, stress was significantly associated with increasing problematic internet use, either through a direct effect (β: 0.43, 95% CI (0.17 to 0.68) or indirectly through depression (β: 0.36, 95% CI (0.2 to 0.54) and anxiety (β: 0.24, 95% CI (0.07 to 0.4)). The overall indirect effect also showed a statistically significant association (β: 0.6, 95% CI (0.44 to 0.77), suggesting partial mediation. See Table 4.

**Table 4:** Key paths and mediation effects of perceived stress on internet and pornography addiction through depression and anxiety.

Path	IAT outcome		PPCS outcome	
	B (95% CI)	*p*	B (95% CI)	*p*
A paths				
Stress → Depression (a1)	0.69 [0.63, 0.76]	**< 0.001**	0.69 [0.63, 0.76]	**< 0.001**
Stress → Anxiety (a2)	0.58 [0.52, 0.63]	**< 0.001**	0.58 [0.52, 0.63]	**< 0.001**
B paths				
Depression → Outcome (b1)	0.53 [0.29, 0.77]	**< 0.001**	0.79 [0.50, 1.11]	**< 0.001**
Anxiety → Outcome (b2)	0.41 [0.13, 0.67]	**0.003**	0.54 [0.20, 0.86]	**0.001**
Direct effect (c’)	0.43 [0.17, 0.68]	**< 0.001**	–0.20 [–0.51, 0.10]	0.196
Indirect effects				
Via depression (a1 × b1)	0.36 [0.20, 0.54]	**< 0.001**	0.55 [0.34, 0.78]	**< 0.001**
Via anxiety (a2 × b2)	0.24 [0.07, 0.40]	**0.004**	0.31 [0.12, 0.50]	**0.002**
Total indirect	0.60 [0.44, 0.77]	**< 0.001**	0.86 [0.66, 1.08]	**< 0.001**
Total effect	1.03 [0.82, 1.25]	**< 0.001**	0.66 [0.40, 0.94]	**< 0.001**

For the problematic pornography use model, the indirect association between stress and problematic pornography use was statistically significant through both depression and anxiety, whereas the direct effect was not statistically significant, suggesting full mediation. See Table 4.

With respect to sleep duration, sleeping less than 6 h was associated with a statistically significant increase in anxiety but not depression. For the problematic internet use model, the association between sleeping less than 6 h and problematic internet use was mediated via only the direct effect rather than the indirect effect. For problematic pornography use, the association was mediated mainly via the indirect effect of anxiety on sleeping, whereas both the total indirect effect and the total effect showed no association between porn and sleeping less than 6 h. See Table 5.

**Table 5:** Key paths and mediation effects of sleep duration on internet and pornography addiction, with depression and anxiety as tested mediators.

Path	IAT outcome		PPCS outcome	
	B (95% CI)	*p*	B (95% CI)	*p*
Less than 6 h vs. 7–8 h				
A path: Sleep < 6 h → Depression (a1_less)	0.33 [− 0.35, 1.01]	0.341	0.33 [− 0.35, 1.01]	0.341
A path: Sleep < 6 h → Anxiety (a2_less)	0.58 [0.02, 1.17]	**0.048**	0.58 [0.02, 1.17]	**0.048**
B paths: Depression → Outcome (b1)	0.53 [0.29, 0.77]	**< 0.001**	0.79 [0.50, 1.10]	**< 0.001**
B paths: Anxiety → Outcome (b2)	0.41 [0.13, 0.67]	**0.003**	0.54 [0.21, 0.87]	**0.002**
Direct effect (c’_less)	− 2.75 [− 4.91, − 0.58]	**0.013**	− 0.83 [− 3.28, 1.72]	0.514
Indirect via depression (a1_less × b1)	0.18 [− 0.19, 0.58]	0.362	0.26 [− 0.29, 0.82]	0.348
Indirect via anxiety (a2_less × b2)	0.24 [0.00, 0.57]	0.113	0.31 [0.01, 0.77]	0.117
Total indirect	0.41 [− 0.14, 1.00]	0.149	0.58 [− 0.21, 1.41]	0.157
Total effect	− 2.33 [− 4.57, − 0.09]	**0.04**	− 0.25 [− 2.77, 2.36]	0.849
More than 9 h vs. 7–8 h				
A path: Sleep > 9 h → Depression (a1_more)	0.83 [− 0.65, 2.38]	0.284	0.83 [− 0.65, 2.38]	0.284
A path: Sleep > 9 h → Anxiety (a2_more)	0.41 [− 0.86, 1.73]	0.523	0.41 [− 0.86, 1.73]	0.523
B paths: Depression → Outcome (b1)	0.53 [0.29, 0.77]	**< 0.001**	0.79 [0.50, 1.10]	**< 0.001**
B paths: Anxiety → Outcome (b2)	0.41 [0.13, 0.67]	**0.003**	0.54 [0.21, 0.87]	**0.002**
Direct effect (c’_more)	0.97 [− 4.62, 6.77]	0.744	7.52 [1.48, 13.84]	**0.018**
Indirect via depression (a1_more × b1)	0.43 [− 0.35, 1.33]	0.308	0.65 [− 0.50, 2.01]	0.304
Indirect via anxiety (a2_more × b2)	0.17 [− 0.37, 0.81]	0.555	0.22 [− 0.47, 1.08]	0.56
Total indirect	0.60 [− 0.58, 1.84]	0.32	0.88 [− 0.77, 2.65]	0.315
Total effect	1.57 [− 4.23, 7.63]	0.604	8.39 [1.75, 15.41]	**0.017**

On the other hand, for those sleeping more than 9 h, no direct or indirect associations were observed in the problematic internet use model, whereas for the problematic pornography use model, the significant associations were mainly direct effects. See Table 5.

## Discussion

Behavioral addiction, including problematic internet use, gaming disorder, and problematic pornography use, is a problematic health problem that affects an increasing population worldwide^35^. Our study aimed to highlight the prevalence of behavioral addiction among medical students in three Arab countries and assess its association with psychological disorders, including stress, anxiety, and depression.

Our findings revealed that among the 1284 participants, 370 had problematic internet use disorder (28.82%). We also found a significant difference in problematic internet use prevalence among the three countries: Egypt had the highest prevalence, at 35%, followed by Sudan, at 27%, and then Libya, at 16%.

Several studies have assessed the prevalence of problematic internet use among several populations, with great variation in their results, ranging from 7.9% to 76.4%^36–41^. A recent multinational cross-sectional study with results that varied from ours was published. Fadl et al.^10^ assessed the prevalence of problematic internet use among adolescents and youth in 26 countries worldwide. They reported that the prevalence of problematic internet use was 38%. They included the three countries that we included; however, they reported that the prevalence in Egypt was 45%, whereas in Libya, it was 50%, and in Sudan, it was 52.5%. This great variation from our results is because Fadl et al. (2025) used a different problematic internet use scale, and they estimated the prevalence of problematic internet use in adolescents and youth; however, we concentrated our results on only medical students to help us better understand their problematic internet use level. Latifeh et al.^16^ conducted a recent cross-sectional study among medical students in Syria. They reported a prevalence of 15.1%. They included all medical, pharmacy, and dental students. Despite their lower prevalence than ours did, they reported that medical students had a prevalence of 34.52%, which was very close to our findings. Finally, Mboya et al.^42^ was another cross-sectional study among medical students and health-allied students in Tanzania. They reported a prevalence of 31%, which was also similar to our results. These results support the trend of increasing problematic internet use levels. It also opens the door to its negative effect on the academic performance of students, as we will discuss in the coming sections.

For gaming disorder, we found a lower prevalence than for problematic internet use. We found that only 6.78% were gaming addicts. Nevertheless, we also found a significant association between country and prevalence, as Egypt had the highest prevalence, at 9.3%, followed by Sudan, at 5.2%, and then Libya, at 2.7%. A previous systematic review assessed the prevalence of gaming disorder; their pooled analysis revealed that the prevalence was approximately 10.1% among the general population^43^. For medical students, several recent studies have shown a lower prevalence than ours. Siste et al.^44^ assessed the prevalence of problematic internet use among Indonesian medical students. They reported that approximately 2.03% were gaming addicts. Finally, Hakami et al.^45^ assessed the prevalence of internet, gaming, and problematic pornography uses among medical students in Saudi Arabia. They reported a high prevalence of problematic internet use (45.8%), whereas the prevalence of gaming disorders was only 2.2%. This variation may be due to the students’ use of smartphones, especially in their studies; for example, in Egypt, medical students rely on smartphones for learning because of the integrated medical system applied in Egypt. This has led medical students in Egypt to use their phones extensively, leading to an increase in their internet and gaming levels. On the other hand, in Sudan and Libya, due to the war, internet access usage has been limited, resulting in universities not relying much on digital learning^46,47^.

Finally, the porn prevalence was approximately 7.55%, and again, there was significant variation among the three countries. Egypt had the highest prevalence, at approximately 9.3%, followed by Sudan, at 6.7%, and Libya, at 4.3%. In Hakami et al.^45^, the prevalence was approximately 11.6%, whereas Karim et al.^13^, who assessed the prevalence of problematic pornography use among nurse students in Egypt, reported a 5.6% addiction level. All these results revealed a significant variance in the population regarding this important topic, making it important to further assess the causes of the increasing prevalence of porn among university students to help manage it.

Psychological disorders have a significant association with behavioral addiction. Our results showed that anxiety, depression, and stress contributed significantly to problematic internet use, gaming disorder, and problematic pornography use. We found that an increase in the anxiety score was significantly positively associated with the problematic internet use score by 0.41, the gaming disorder score by approximately 0.22, and the Problematic pornography use score by approximately 0.53. Depression had a similar association, as its increase was significantly positively associated with an increase in the problematic internet use score by approximately 0.52, gaming disorder by approximately 0.26, and problematic pornography use by approximately 0.8. Finally, stress had different results, as its increase was positively associated with the problematic internet use of approximately 0.45, whereas it was negatively associated with the gaming disorder and problematic pornography use of approximately 0.17 and 0.54, respectively. The results of the mediation analysis further confirmed the association with stress level, as, in terms of the direct effect, stress significantly increased problematic internet use and significantly decreased problematic pornography use, whereas the indirect effect showed that stress increased both depression and anxiety, which significantly increased problematic internet use. Despite the similarity of our results for depression and anxiety with those of previous literature^48,49^, as we will discuss in the next paragraph, the association with stress contradicts the literature that suggests a significant association between behavioral addiction and stress. However, the reason for our findings is not fully understood, and several studies are needed to further assess this relationship. Overall, a possible explanation is the distribution of the students in the current study, as more than half of the students are from fourth years to interns, and a previous study among medical students conducted in Uganda revealed that internet and smartphone addiction increases with increasing academic year, which can be explained by the coping strategies of older medical students in the university curriculum^50^. In our study, a high percentage of older medical students were present, so due to their coping strategies with the university curriculum, their anxiety level may not affect their gaming disorder or problematic pornography because they usually require great cognitive engagement; however, it can easily increase the level of problematic internet use, which usually does not require the cognitive engagement required in gaming disorder and problematic pornography use. On the other hand, as a cross-sectional study, the results are not conclusive and may be affected by several confounders, so several studies are still needed to further assess these results.

Several studies have shown similar results; for example, Karim et al.^13^ reported a weak positive correlation between problematic pornography use and all three psychological disorders (stress, anxiety, and depression). Hakami et al.^45^ also reported a weak correlation between three types of behavioral addictions and three types of psychological disorders. In 2021, Siste et al.^44^. reported no significant associations between gaming disorder and both depression and anxiety. Their logistic regression model revealed that increasing the depression score was associated with increasing the odds of being a gaming addict by approximately 1.62, whereas increasing the anxiety score was associated with decreasing the odds by approximately 0.35 (P values: 0.73 and 0.68, respectively). This variation between our results and theirs may be due to the small sample size of the Siste study, their smaller prevalence than ours, or the fact that they may not use smartphones frequently in their learning methods.

Previous studies have shown a significant association between a reduction in the level of problematic internet use and an increase in age, and the population aged 21–25 years has the highest level of problematic internet use^51^. Our results did not reveal a statistically significant association between age and any of the behavioral addictions measured. This may be because all the students were aged 18–25 years. This finding was consistent with those of previous studies^13,42,44,45,52^. We also did not find any association between gender and behavioral addiction, which indicates that both genders can be affected equally by this type of addiction. This finding was also consistent with those of previous studies^13,42,44,45,52^.

There was no association between family income and behavioral addiction levels. Previous studies have shown that low-income countries have a higher prevalence than high-income countries do^10,53^. They showed that this variation occurs because low-income counties face much more stress than high-income counties do. In our study, we did not find this association because all three countries are low-income countries. Additionally, we revealed that rural residents had higher problematic internet use levels than urban residents. This result again differed from those of previous literature, which showed that urban residents had more problematic internet use than did rural residents^10,39^. This variation occurs because all the participants in our study are medical students, who visit their universities more frequently than other specialists do, and their universities are often located in cities. This makes rural residents travel to their universities more frequently than urban students do, who often live near their universities. This frequent travel makes students much more likely to use their smartphones during their travel than other students are, making them more likely to be internet addicts.

Interestingly, we did not find an association between behavioral addiction and students’ academic performance, whereas we found a statistically significant negative association between behavioral addiction and satisfaction with academic performance. The results revealed that the highest level of internet and problematic pornography use was found among students who were very unsatisfied with their academic performance. The same findings were observed in terms of life satisfaction, as the highest levels of internet, gaming, and problematic pornography uses were found in very unsatisfied students. Latifeh et al.^16^ reported that the highest level of problematic internet use was found in students with the lowest academic degree. They did not include a satisfaction question, so we are not sure if their results are consistent with ours or not.

The linear regression results revealed a significant association between behavioral addiction and sleeping hours. The results revealed that sleeping less than 6 h was associated with a significant reduction in the level of problematic internet use. We also found a positive association between sleeping more than 9 h and increasing both gaming and problematic pornography use. Furthermore, the mediation analysis revealed no indirect effect of sleeping on internet or problematic pornography use, whereas the effect was only direct. Several studies have demonstrated an association between poor sleep quality and either psychological disorders or smartphone and problematic internet use disorders^54–56^. The current results concerning sleeping for more than 9 h may not reflect healthy sleep patterns but may reflect disturbed sleeping patterns, as previous studies revealed a significant association between social media fatigue, or addiction, and delayed sleeping hours, leading to disturbances in circadian rhythm and daily fatigue^57^. Nevertheless, we cannot support this hypothesis in our study because we did not use a validated questionnaire to assess sleep quality because of the large questionnaire. Overall, several studies are needed to further assess these results to better understand the exact associations between these addictions and sleeping hours.

This may be explained by the fact that the internet, or a porn addict, may withdraw from students’ daily activities or duties, such as studying, leading to an increase in their sleeping hours, whereas the students who study hard usually sleep less, which leads to a reduction in their internet and problematic pornography uses.

### Strengths and limitations

Our study has several strengths. We included only medical students, so we can concentrate our results on this important population. The inclusion of three behavioral addictions allows us to fully understand the interaction between these three variables and to better understand the factors associated with their increase. Additionally, we include three countries that are Arabic and share similar cultures, making our results informative and representative of African Arab countries.

On the other hand, several limitations should be noted. We use a cross-sectional study with a convenience sampling technique for each country, which can lead to selection bias, limiting the study’s generalizability. Additionally, we included problematic pornography use, which can prevent several students from completing the questionnaire or reporting untrue results due to cultural considerations. This can lead to bias in the actual results. Additionally, in Libya, we could not collect the total sample size required because of the poor response rate. Finally, the questionnaire was long, which also made many students unwilling to fill it out.

## Conclusions

Our cross-sectional study assessed the prevalence of three types of behavioral addictions (Internet, gaming, and problematic pornography use) among medical students in three Arab and North African countries (Egypt, Sudan, and Libya). We found that the prevalence rates were 28.82%, 6.78%, and 7.55% for internet, gaming, and Problematic pornography uses, respectively. We found a significant association between increasing behavioral addiction and psychological disorders, including stress, anxiety, and depression. No difference was observed between the two genders, whereas rural residents reported greater problematic internet use. Increased satisfaction was also associated with reduced behavioral addictions, whereas increased sleeping hours were positively associated with increased addiction.

## Supplementary Information

Below is the link to the electronic supplementary material.

Supplementary Material 1

## Data Availability

All data are provided in the manuscript and its related supplementary material.

## Ddeclarations

### Artificial intelligence usage

The author did not use any artificial intelligence at any step of the study process.
