# Supplementary material for: Association between behavioral addiction and psychological disorders among medical students in Egypt, Sudan, and Libya: a cross-sectional study
Source: Sci Rep. 2026 Jun 4;16:17375. doi: 10.1038/s41598-026-56057-9 (PMC13237368; doi:10.1038/s41598-026-56057-9)

**Supplementary material**

Table of Contents

[**Table S1**: reliability of the used questionnaire (McDonald’s Omega) 2](#_Toc229126640)

[**Table S2: comparison of** absolute prevalence by country 2](#_Toc229126641)

[**Table S3: Comparison of** absolute prevalence by Gender 3](#_Toc229126642)

[**Table S4: comparison of** absolute prevalence by University type 3](#_Toc229126643)

[**Table S5: comparison of** absolute prevalence by Study Phase 4](#_Toc229126644)

[**Table S6: comparison of** absolute prevalence by monthly income 4](#_Toc229126645)

[**Table S7: Comparison of** absolute prevalence by Residency 5](#_Toc229126646)

[**Table S8: Comparison of** absolute prevalence by Living situation 5](#_Toc229126647)

[**Table S9: comparison of** absolute prevalence by Smoking status 6](#_Toc229126648)

[**Table S10: comparison of** absolute prevalence by Family history of mental diseases 6](#_Toc229126649)

[**Table S11: Comparison of** absolute prevalence by Academic grade 7](#_Toc229126650)

[**Table S12: comparison of** absolute prevalence by history of chronic disease 7](#_Toc229126651)

[**Table S13: Comparison of** absolute prevalence by Satisfaction with academic grade 8](#_Toc229126652)

[**Table S14: comparison of** absolute prevalence by Life satisfaction. 8](#_Toc229126653)

[**Table S15: Comparison of** absolute prevalence by Exercise frequency 9](#_Toc229126654)

[**Table S16:** collinearity assessment for the internet addiction and gaming addiction 9](#_Toc229126655)

[**Table S17:** collinearity assessment for porn addiction scale 10](#_Toc229126656)

[**Table S18:** fit indices of the SEM mediation models 10](#_Toc229126657)

[**Figure S19:** Full parameter estimates for the parallel dual-mediator structural equation model with pornography addiction (PPCS score) as the outcome: paths through depression (PHQ-9) and anxiety (GAD-7), with perceived stress (PSS) as the exposure. Bootstrap standard errors based on 5,000 resamples (N = 1,284). 11](#_Toc229126658)

[**Table S20.** Full parameter estimates for the parallel dual-mediator structural equation model with internet addiction (IAT score) as the outcome: paths through depression (PHQ-9) and anxiety (GAD-7), with perceived stress (PSS) as the exposure. Bootstrap standard errors based on 5,000 resamples (N = 1,284). 14](#_Toc229126659)

[**Table S21.** Full parameter estimates for the parallel dual-mediator structural equation model with pornography addiction (PPCS score) as the outcome: paths through depression (PHQ-9) and anxiety (GAD-7), with sleep duration as the exposure and perceived stress (PSS) as a covariate. Bootstrap standard errors based on 5,000 resamples (N = 1,284). 17](#_Toc229126660)

[**Table S22.** Full parameter estimates for the parallel dual-mediator structural equation model with internet addiction (IAT score) as the outcome: paths through depression (PHQ-9) and anxiety (GAD-7), with sleep duration as the exposure and perceived stress (PSS) as a covariate. Bootstrap standard errors based on 5,000 resamples (N = 1,284). 21](#_Toc229126661)

[**Figure S1:** Histogram and Q-Q plot for age with normality test results 26](#_Toc229126662)

[**Figure S2**: IAT individual items Likert plot 27](#_Toc229126663)

[**Figure S3:** PPCS individual items Likert plot 29](#_Toc229126664)

[**Figure S4:** IGDS individual items Likert plot 30](#_Toc229126665)

[**Figure S5**: Density plot represent the latent profile analysis 31](#_Toc229126666)

# **Table S1**: reliability of the used questionnaire (McDonald’s Omega)

| **Scale** | **McDonald’s Ω (Total)** |
| --- | --- |
| Internet Addiction Test (IAT) | 0.95 |
| Internet Gaming Disorder Scale (IGDS) | 0.95 |
| Problematic Pornography Consumption Scale (PPCS) | 0.98 |
| Patient Health Questionnaire (PHQ-9) | 0.91 |
| Generalized Anxiety Disorder Scale (GAD-7) | 0.92 |
| Perceived Stress Scale (PSS) | 0.93 |

# **Table S2: comparison of** absolute prevalence by country

| **variable** | **Egypt** | | **Libya** | | **Sudan** | | **p-value***^3^* |
| --- | --- | --- | --- | --- | --- | --- | --- |
|  | **N = 616*^1^*** | **95% CI*^2^*** | **N = 187*^1^*** | **95% CI*^2^*** | **N = 481*^1^*** | **95% CI*^2^*** |  |
| PHQ-9 (cut-off ≥ 10) | 341 (55%) | 51%, 59% | 67 (36%) | 29%, 43% | 196 (41%) | 36%, 45% | **<0.001** |
| GAD-7 (cut-off ≥ 10) | 279 (45%) | 41%, 49% | 63 (34%) | 27%, 41% | 148 (31%) | 27%, 35% | **<0.001** |
| IAT (cut-off ≥ 50) | 213 (35%) | 31%, 39% | 29 (16%) | 11%, 22% | 128 (27%) | 23%, 31% | **<0.001** |
| PSS (cut-off ≥ 27) | 69 (11%) | 8.9%, 14% | 8 (4.3%) | 2.0%, 8.6% | 28 (5.8%) | 4.0%, 8.4% | **<0.001** |
| PPCS (cut-off ≥ 76) | 57 (9.3%) | 7.1%, 12% | 8 (4.3%) | 2.0%, 8.6% | 32 (6.7%) | 4.7%, 9.4% | 0.05 |
| IGDS (cut-off ≥ 32) | 57 (9.3%) | 7.1%, 12% | 5 (2.7%) | 0.99%, 6.5% | 25 (5.2%) | 3.5%, 7.7% | **0.002** |
| *^1^ n (%) ^2^ CI = Confidence Interval calculated using the Wilson method ^3^ Pearson’s Chi-squared test* | | | | | | | |

# **Table S3: Comparison of** absolute prevalence by Gender

| **Characteristic** | **Female** | **95% CI***^2^* | **Male** | **95% CI***^2^* | **p-value***^3^* |
| --- | --- | --- | --- | --- | --- |
|  | N = 795*^1^* |  | N = 489*^1^* |  |  |
| PHQ-9 (cut-off ≥ 10) | 376 (47%) | 44%, 51% | 228 (47%) | 42%, 51% | 0.8 |
| GAD-7 (cut-off ≥ 10) | 314 (39%) | 36%, 43% | 176 (36%) | 32%, 40% | 0.2 |
| IAT (cut-off ≥ 50) | 231 (29%) | 26%, 32% | 139 (28%) | 25%, 33% | 0.8 |
| PSS (cut-off ≥ 27) | 70 (8.8%) | 7.0%, 11% | 35 (7.2%) | 5.1%, 9.9% | 0.3 |
| PPCS (cut-off ≥ 76) | 40 (5.0%) | 3.7%, 6.9% | 57 (12%) | 9.0%, 15% | **<0.001** |
| IGDS (cut-off ≥ 32) | 45 (5.7%) | 4.2%, 7.6% | 42 (8.6%) | 6.3%, 12% | **0.043** |
| *^1^* n (%) | | | | | |
| *^2^* CI = Confidence Interval | | | | | |
| *^3^* Pearson’s Chi-squared test | | | | | |

# **Table S4: comparison of** absolute prevalence by University type

| **Characteristic** | **National** | **95% CI***^2^* | **Private** | **95% CI***^2^* | **Public** | **95% CI***^2^* | **p-value***^3^* |  |
| --- | --- | --- | --- | --- | --- | --- | --- | --- |
|  | N = 79*^1^* |  | N = 185*^1^* |  | N = 1,020*^1^* |  |  |  |
| PHQ-9 (cut-off ≥ 10) | 38 (48%) | 37%, 60% | 93 (50%) | 43%, 58% | 473 (46%) | 43%, 49% | 0.6 |  |
| GAD-7 (cut-off ≥ 10) | 28 (35%) | 25%, 47% | 67 (36%) | 29%, 44% | 395 (39%) | 36%, 42% | 0.7 |  |
| IAT (cut-off ≥ 50) | 28 (35%) | 25%, 47% | 42 (23%) | 17%, 30% | 300 (29%) | 27%, 32% | 0.073 |  |
| PSS (cut-off ≥ 27) | 9 (11%) | 5.7%, 21% | 14 (7.6%) | 4.4%, 13% | 82 (8.0%) | 6.5%, 9.9% | 0.5 |  |
| PPCS (cut-off ≥ 76) | 6 (7.6%) | 3.1%, 16% | 17 (9.2%) | 5.6%, 15% | 74 (7.3%) | 5.8%, 9.1% | 0.7 |  |
| IGDS (cut-off ≥ 32) | 9 (11%) | 5.7%, 21% | 12 (6.5%) | 3.5%, 11% | 66 (6.5%) | 5.1%, 8.2% | 0.2 |  |
| *^1^* n (%) | | | | | | | | |
| *^2^* CI = Confidence Interval | | | | | | | | |

# **Table S5: comparison of** absolute prevalence by Study Phase

| **Characteristic** | **Academic phase** | **95% CI***^2^* | **Clinical phase** | **95% CI***^2^* | **Intern** | **95% CI***^2^* | **p-value***^3^* |
| --- | --- | --- | --- | --- | --- | --- | --- |
|  | N = 480*^1^* |  | N = 590*^1^* |  | N = 214*^1^* |  |  |
| PHQ-9 (cut-off ≥ 10) | 227 (47%) | 43%, 52% | 291 (49%) | 45%, 53% | 86 (40%) | 34%, 47% | 0.071 |
| GAD-7 (cut-off ≥ 10) | 174 (36%) | 32%, 41% | 240 (41%) | 37%, 45% | 76 (36%) | 29%, 42% | 0.2 |
| IAT (cut-off ≥ 50) | 146 (30%) | 26%, 35% | 172 (29%) | 26%, 33% | 52 (24%) | 19%, 31% | 0.3 |
| PSS (cut-off ≥ 27) | 45 (9.4%) | 7.0%, 12% | 41 (6.9%) | 5.1%, 9.4% | 19 (8.9%) | 5.6%, 14% | 0.3 |
| PPCS (cut-off ≥ 76) | 44 (9.2%) | 6.8%, 12% | 40 (6.8%) | 4.9%, 9.2% | 13 (6.1%) | 3.4%, 10% | 0.2 |
| IGDS (cut-off ≥ 32) | 31 (6.5%) | 4.5%, 9.1% | 40 (6.8%) | 4.9%, 9.2% | 16 (7.5%) | 4.5%, 12% | 0.9 |
| *^1^* n (%) | | | | | | | |
| *^2^* CI = Confidence Interval | | | | | | | |
| *^3^* Pearson’s Chi-squared test | | | | | | | |

# **Table S6: comparison of** absolute prevalence by monthly income

| **Characteristic** | **Enough and saving** | **95% CI***^2^* | **Enough, but not saving** | **95% CI***^2^* | **Not enough** | **95% CI***^2^* | **p-value***^3^* |  |
| --- | --- | --- | --- | --- | --- | --- | --- | --- |
|  | N = 555*^1^* |  | N = 620*^1^* |  | N = 109*^1^* |  |  |  |
| PHQ-9 (cut-off ≥ 10) | 231 (42%) | 38%, 46% | 315 (51%) | 47%, 55% | 58 (53%) | 43%, 63% | **0.003** |  |
| GAD-7 (cut-off ≥ 10) | 187 (34%) | 30%, 38% | 260 (42%) | 38%, 46% | 43 (39%) | 30%, 49% | **0.014** |  |
| IAT (cut-off ≥ 50) | 157 (28%) | 25%, 32% | 182 (29%) | 26%, 33% | 31 (28%) | 20%, 38% | >0.9 |  |
| PSS (cut-off ≥ 27) | 46 (8.3%) | 6.2%, 11% | 45 (7.3%) | 5.4%, 9.7% | 14 (13%) | 7.5%, 21% | 0.14 |  |
| PPCS (cut-off ≥ 76) | 42 (7.6%) | 5.6%, 10% | 45 (7.3%) | 5.4%, 9.7% | 10 (9.2%) | 4.7%, 17% | 0.8 |  |
| IGDS (cut-off ≥ 32) | 35 (6.3%) | 4.5%, 8.7% | 43 (6.9%) | 5.1%, 9.3% | 9 (8.3%) | 4.1%, 16% | 0.7 |  |
| *^1^* n (%) | | | | | | | | |
| *^2^* CI = Confidence Interval | | | | | | | | |
| *^3^* Pearson’s Chi-squared test | | | | | | | | |

# **Table S7: Comparison of** absolute prevalence by Residency

| **Characteristic** | **Rural** | **95% CI***^2^* | **Urban** | **95% CI***^2^* | **p-value***^3^* |
| --- | --- | --- | --- | --- | --- |
|  | N = 480*^1^* |  | N = 804*^1^* |  |  |
| PHQ-9 (cut-off ≥ 10) | 234 (49%) | 44%, 53% | 370 (46%) | 43%, 50% | 0.3 |
| GAD-7 (cut-off ≥ 10) | 177 (37%) | 33%, 41% | 313 (39%) | 36%, 42% | 0.5 |
| IAT (cut-off ≥ 50) | 155 (32%) | 28%, 37% | 215 (27%) | 24%, 30% | **0.034** |
| PSS (cut-off ≥ 27) | 33 (6.9%) | 4.8%, 9.6% | 72 (9.0%) | 7.1%, 11% | 0.2 |
| PPCS (cut-off ≥ 76) | 39 (8.1%) | 5.9%, 11% | 58 (7.2%) | 5.6%, 9.3% | 0.6 |
| IGDS (cut-off ≥ 32) | 40 (8.3%) | 6.1%, 11% | 47 (5.8%) | 4.4%, 7.8% | 0.086 |
| *^1^* n (%) | | | | | |
| *^2^* CI = Confidence Interval | | | | | |
| *^3^* Pearson’s Chi-squared test | | | | | |

# **Table S8: Comparison of** absolute prevalence by Living situation

| **Characteristic** | **Living alone** | **95% CI***^2^* | **Living with family** | **95% CI***^2^* | **Living with friends** | **95% CI***^2^* | **p-value***^3^* |  |
| --- | --- | --- | --- | --- | --- | --- | --- | --- |
|  | N = 61*^1^* |  | N = 922*^1^* |  | N = 301*^1^* |  |  |  |
| PHQ-9 (cut-off ≥ 10) | 36 (59%) | 46%, 71% | 435 (47%) | 44%, 50% | 133 (44%) | 39%, 50% | 0.11 |  |
| GAD-7 (cut-off ≥ 10) | 35 (57%) | 44%, 70% | 349 (38%) | 35%, 41% | 106 (35%) | 30%, 41% | 0.005 |  |
| PSS (cut-off ≥ 27) | 8 (13%) | 6.2%, 25% | 81 (8.8%) | 7.1%, 11% | 16 (5.3%) | 3.2%, 8.7% | **0.049** |  |
| IAT (cut-off ≥ 50) | 12 (20%) | 11%, 32% | 262 (28%) | 26%, 31% | 96 (32%) | 27%, 38% | 0.14 |  |
| PPCS (cut-off ≥ 76) | 8 (13%) | 6.2%, 25% | 66 (7.2%) | 5.6%, 9.1% | 23 (7.6%) | 5.0%, 11% | 0.2 |  |
| IGDS (cut-off ≥ 32) | 1 (1.6%) | 0.09%, 10% | 65 (7.0%) | 5.5%, 8.9% | 21 (7.0%) | 4.5%, 11% | 0.3 |  |
| *^1^* n (%) | | | | | | | | |
| *^2^* CI = Confidence Interval | | | | | | | | |
| *^3^* Pearson’s Chi-squared test; Fisher’s exact test | | | | | | | | |

# **Table S9: comparison of** absolute prevalence by Smoking status

| **Characteristic** | **No** | **95% CI***^2^* | **Yes** | **95% CI***^2^* | **p-value***^3^* |
| --- | --- | --- | --- | --- | --- |
|  | N = 1,226*^1^* |  | N = 58*^1^* |  |  |
| PHQ-9 (cut-off ≥ 10) | 572 (47%) | 44%, 49% | 32 (55%) | 42%, 68% | 0.2 |
| GAD-7 (cut-off ≥ 10) | 457 (37%) | 35%, 40% | 33 (57%) | 43%, 70% | **0.003** |
| IAT (cut-off ≥ 50) | 356 (29%) | 27%, 32% | 14 (24%) | 14%, 37% | 0.4 |
| PSS (cut-off ≥ 27) | 97 (7.9%) | 6.5%, 9.6% | 8 (14%) | 6.6%, 26% | 0.13 |
| PPCS (cut-off ≥ 76) | 87 (7.1%) | 5.8%, 8.7% | 10 (17%) | 9.0%, 30% | **0.009** |
| IGDS (cut-off ≥ 32) | 85 (6.9%) | 5.6%, 8.5% | 2 (3.4%) | 0.60%, 13% | 0.4 |
| *^1^* n (%) | | | | | |
| *^2^* CI = Confidence Interval | | | | | |
| *^3^* Pearson’s Chi-squared test; Fisher’s exact test | | | | | |

# **Table S10: comparison of** absolute prevalence by Family history of mental diseases

| **Characteristic** | **No** | **95% CI***^2^* | **Yes** | **95% CI***^2^* | **p-value***^3^* |
| --- | --- | --- | --- | --- | --- |
|  | N = 1,120*^1^* |  | N = 164*^1^* |  |  |
| PHQ-9 (cut-off ≥ 10) | 519 (46%) | 43%, 49% | 85 (52%) | 44%, 60% | 0.2 |
| GAD-7 (cut-off ≥ 10) | 417 (37%) | 34%, 40% | 73 (45%) | 37%, 52% | 0.073 |
| IAT (cut-off ≥ 50) | 322 (29%) | 26%, 32% | 48 (29%) | 23%, 37% | 0.9 |
| PSS (cut-off ≥ 27) | 83 (7.4%) | 6.0%, 9.1% | 22 (13%) | 8.8%, 20% | **0.009** |
| PPCS (cut-off ≥ 76) | 81 (7.2%) | 5.8%, 8.9% | 16 (9.8%) | 5.9%, 16% | 0.3 |
| IGDS (cut-off ≥ 32) | 75 (6.7%) | 5.3%, 8.4% | 12 (7.3%) | 4.0%, 13% | 0.8 |
| *^1^* n (%) | | | | | |
| *^2^* CI = Confidence Interval | | | | | |
| *^3^* Pearson’s Chi-squared test | | | | | |

# **Table S11: Comparison of** absolute prevalence by Academic grade

| **Characteristic** | **Excellent** | **95% CI***^2^* | **Very good** | **95% CI***^2^* | **Good** | **95% CI***^2^* | **Fair** | **95% CI***^2^* | **Failed** | **95% CI***^2^* | **p-value***^3^* |
| --- | --- | --- | --- | --- | --- | --- | --- | --- | --- | --- | --- |
|  | N = 459*^1^* |  | N = 424*^1^* |  | N = 350*^1^* |  | N = 43*^1^* |  | N = 8*^1^* |  |  |
| PHQ-9 (cut-off ≥ 10) | 231 (50%) | 46%, 55% | 188 (44%) | 40%, 49% | 157 (45%) | 40%, 50% | 24 (56%) | 40%, 71% | 4 (50%) | 22%, 78% | 0.3 |
| GAD-7 (cut-off ≥ 10) | 181 (39%) | 35%, 44% | 155 (37%) | 32%, 41% | 130 (37%) | 32%, 42% | 19 (44%) | 29%, 60% | 5 (63%) | 26%, 90% | 0.5 |
| IAT (cut-off ≥ 50) | 131 (29%) | 24%, 33% | 130 (31%) | 26%, 35% | 90 (26%) | 21%, 31% | 15 (35%) | 21%, 51% | 4 (50%) | 22%, 78% | 0.3 |
| PSS (cut-off ≥ 27) | 40 (8.7%) | 6.4%, 12% | 34 (8.0%) | 5.7%, 11% | 19 (5.4%) | 3.4%, 8.5% | 9 (21%) | 11%, 36% | 3 (38%) | 10%, 74% | **<0.001** |
| PPCS (cut-off ≥ 76) | 35 (7.6%) | 5.4%, 11% | 31 (7.3%) | 5.1%, 10% | 22 (6.3%) | 4.1%, 9.5% | 7 (16%) | 7.3%, 31% | 2 (25%) | 4.5%, 64% | 0.066 |
| IGDS (cut-off ≥ 32) | 38 (8.3%) | 6.0%, 11% | 27 (6.4%) | 4.3%, 9.2% | 16 (4.6%) | 2.7%, 7.5% | 5 (12%) | 4.4%, 26% | 1 (13%) | 0.66%, 53% | 0.1 |
| *^1^* n (%) | | | | | | | | | | | |
| *^2^* CI = Confidence Interval | | | | | | | | | | | |
| *^3^* Fisher’s exact test | | | | | | | | | | | |

# **Table S12: comparison of** absolute prevalence by history of chronic disease

| **Characteristic** | **No** | **95% CI***^2^* | **Yes** | **95% CI***^2^* | **p-value***^3^* |
| --- | --- | --- | --- | --- | --- |
|  | N = 1,153*^1^* |  | N = 131*^1^* |  |  |
| PHQ-9 (cut-off ≥ 10) | 536 (46%) | 44%, 49% | 68 (52%) | 43%, 61% | 0.2 |
| GAD-7 (cut-off ≥ 10) | 435 (38%) | 35%, 41% | 55 (42%) | 34%, 51% | 0.3 |
| IAT (cut-off ≥ 50) | 334 (29%) | 26%, 32% | 36 (27%) | 20%, 36% | 0.7 |
| PSS (cut-off ≥ 27) | 90 (7.8%) | 6.4%, 9.5% | 15 (11%) | 6.8%, 18% | 0.15 |
| PPCS (cut-off ≥ 76) | 90 (7.8%) | 6.4%, 9.5% | 7 (5.3%) | 2.4%, 11% | 0.3 |
| IGDS (cut-off ≥ 32) | 78 (6.8%) | 5.4%, 8.4% | 9 (6.9%) | 3.4%, 13% | >0.9 |
| *^1^* n (%) | | | | | |
| *^2^* CI = Confidence Interval | | | | | |
| *^3^* Pearson’s Chi-squared test | | | | | |

# **Table S13: Comparison of** absolute prevalence by Satisfaction with academic grade

| **Characteristic** | **Very satisfied** | **95% CI***^2^* | **Satisfied.** | **95% CI***^2^* | **Natural** | **95% CI***^2^* | **unsatisfied** | **95% CI***^2^* | **Very unsatisfied** | **95% CI***^2^* | **p-value***^3^* |
| --- | --- | --- | --- | --- | --- | --- | --- | --- | --- | --- | --- |
|  | N = 156*^1^* |  | N = 466*^1^* |  | N = 405*^1^* |  | N = 208*^1^* |  | N = 49*^1^* |  |  |
| PHQ-9 (cut-off ≥ 10) | 58 (37%) | 30%, 45% | 196 (42%) | 38%, 47% | 201 (50%) | 45%, 55% | 119 (57%) | 50%, 64% | 30 (61%) | 46%, 74% | **<0.001** |
| GAD-7 (cut-off ≥ 10) | 51 (33%) | 26%, 41% | 160 (34%) | 30%, 39% | 157 (39%) | 34%, 44% | 93 (45%) | 38%, 52% | 29 (59%) | 44%, 73% | **0.001** |
| IAT (cut-off ≥ 50) | 33 (21%) | 15%, 29% | 122 (26%) | 22%, 30% | 115 (28%) | 24%, 33% | 79 (38%) | 31%, 45% | 21 (43%) | 29%, 58% | **<0.001** |
| PSS (cut-off ≥ 27) | 6 (3.8%) | 1.6%, 8.6% | 28 (6.0%) | 4.1%, 8.7% | 21 (5.2%) | 3.3%, 7.9% | 33 (16%) | 11%, 22% | 17 (35%) | 22%, 50% | **<0.001** |
| PPCS (cut-off ≥ 76) | 12 (7.7%) | 4.2%, 13% | 18 (3.9%) | 2.4%, 6.2% | 39 (9.6%) | 7.0%, 13% | 19 (9.1%) | 5.7%, 14% | 9 (18%) | 9.2%, 33% | **<0.001** |
| IGDS (cut-off ≥ 32) | 9 (5.8%) | 2.8%, 11% | 25 (5.4%) | 3.6%, 7.9% | 28 (6.9%) | 4.7%, 10% | 18 (8.7%) | 5.4%, 14% | 7 (14%) | 6.4%, 28% | 0.13 |
| *^1^* n (%) | | | | | | | | | | | |
| *^2^* CI = Confidence Interval | | | | | | | | | | | |
| *^3^* Pearson’s Chi-squared test; Fisher’s exact test | | | | | | | | | | | |

# **Table S14: comparison of** absolute prevalence by Life satisfaction.

| **Characteristic** | **Very unsatisfied** | **95% CI***^2^* | **unsatisfied** | **95% CI***^2^* | **Natural** | **95% CI***^2^* | **Satisfied** | **95% CI***^2^* | **Very Satisfied** | **95% CI***^2^* | **p-value***^3^* |  |
| --- | --- | --- | --- | --- | --- | --- | --- | --- | --- | --- | --- | --- |
|  | N = 29*^1^* |  | N = 134*^1^* |  | N = 438*^1^* |  | N = 449*^1^* |  | N = 234*^1^* |  |  |  |
| PHQ-9 (cut-off ≥ 10) | 24 (83%) | 64%, 93% | 91 (68%) | 59%, 76% | 229 (52%) | 47%, 57% | 178 (40%) | 35%, 44% | 82 (35%) | 29%, 42% | **<0.001** |  |
| GAD-7 (cut-off ≥ 10) | 22 (76%) | 56%, 89% | 79 (59%) | 50%, 67% | 189 (43%) | 38%, 48% | 145 (32%) | 28%, 37% | 55 (24%) | 18%, 30% | **<0.001** |  |
| IAT (cut-off ≥ 50) | 13 (45%) | 27%, 64% | 63 (47%) | 38%, 56% | 131 (30%) | 26%, 34% | 120 (27%) | 23%, 31% | 43 (18%) | 14%, 24% | **<0.001** |  |
| PSS (cut-off ≥ 27) | 14 (48%) | 30%, 67% | 29 (22%) | 15%, 30% | 36 (8.2%) | 5.9%, 11% | 20 (4.5%) | 2.8%, 6.9% | 6 (2.6%) | 1.0%, 5.8% | **<0.001** |  |
| PPCS (cut-off ≥ 76) | 6 (21%) | 8.7%, 40% | 18 (13%) | 8.4%, 21% | 39 (8.9%) | 6.5%, 12% | 23 (5.1%) | 3.3%, 7.7% | 11 (4.7%) | 2.5%, 8.5% | **<0.001** |  |
| IGDS (cut-off ≥ 32) | 6 (21%) | 8.7%, 40% | 17 (13%) | 7.8%, 20% | 34 (7.8%) | 5.5%, 11% | 21 (4.7%) | 3.0%, 7.2% | 9 (3.8%) | 1.9%, 7.4% | **<0.001** |  |
| *^1^* n (%) | | | | | | | | | | | | |
| *^2^* CI = Confidence Interval | | | | | | | | | | | | |
| *^3^* Pearson’s Chi-squared test; Fisher’s exact test | | | | | | | | | | | | |

# **Table S15: Comparison of** absolute prevalence by Exercise frequency

| **Characteristic** | **None** | **95% CI***^2^* | **Low** | **95% CI***^2^* | **Moderate** | **95% CI***^2^* | **High** | **95% CI***^2^* | **p-value***^3^* |  |
| --- | --- | --- | --- | --- | --- | --- | --- | --- | --- | --- |
|  | N = 474*^1^* |  | N = 443*^1^* |  | N = 317*^1^* |  | N = 50*^1^* |  |  |  |
| PHQ-9 (cut-off ≥ 10) | 221 (47%) | 42%, 51% | 225 (51%) | 46%, 56% | 129 (41%) | 35%, 46% | 29 (58%) | 43%, 72% | **0.018** |  |
| GAD-7 (cut-off ≥ 10) | 185 (39%) | 35%, 44% | 172 (39%) | 34%, 44% | 114 (36%) | 31%, 42% | 19 (38%) | 25%, 53% | 0.8 |  |
| IAT (cut-off ≥ 50) | 154 (32%) | 28%, 37% | 123 (28%) | 24%, 32% | 85 (27%) | 22%, 32% | 8 (16%) | 7.6%, 30% | **0.046** |  |
| PSS (cut-off ≥ 27) | 46 (9.7%) | 7.3%, 13% | 41 (9.3%) | 6.8%, 12% | 16 (5.0%) | 3.0%, 8.2% | 2 (4.0%) | 0.70%, 15% | 0.056 |  |
| PPCS (cut-off ≥ 76) | 36 (7.6%) | 5.4%, 10% | 33 (7.4%) | 5.3%, 10% | 24 (7.6%) | 5.0%, 11% | 4 (8.0%) | 2.6%, 20% | >0.9 |  |
| IGDS (cut-off ≥ 32) | 34 (7.2%) | 5.1%, 10% | 33 (7.4%) | 5.3%, 10% | 18 (5.7%) | 3.5%, 9.0% | 2 (4.0%) | 0.70%, 15% | 0.7 |  |
| *^1^* n (%) | | | | | | | | | | |
| *^2^* CI = Confidence Interval | | | | | | | | | | |
| *^3^* Pearson’s Chi-squared test; Fisher’s exact test | | | | | | | | | | |

# **Table S16:** collinearity assessment for the internet addiction and gaming addiction

| **IAT** | | | | | **IGDS** | | | | |
| --- | --- | --- | --- | --- | --- | --- | --- | --- | --- |
|  |  |  |  |  |  |  |  |  |  |
| **predictor** | **GVIF** | **Df** | **GVIF^(1/(2*Df))** | **Interacts With** | **predictor** | **GVIF** | **Df** | **GVIF^(1/(2*Df))** | **Interacts With** |
| gad7_score | 2.13 | 1 | 1.46 | -- | gad7_score | 2.13 | 1 | 1.46 | -- |
| phq9_score | 2.13 | 1 | 1.46 | -- | phq9_score | 2.13 | 1 | 1.46 | -- |
| pss_score | 1.55 | 3 | 1.08 | gender | pss_score | 1.55 | 3 | 1.08 | gender |
| gender | 1.55 | 3 | 1.08 | pss_score | gender | 1.55 | 3 | 1.08 | pss_score |
| age | 1.27 | 1 | 1.13 | -- | age | 1.27 | 1 | 1.13 | -- |
| study_country | 1.36 | 2 | 1.08 | -- | study_country | 1.36 | 2 | 1.08 | -- |
| income | 1.05 | 2 | 1.01 | -- | income | 1.05 | 2 | 1.01 | -- |
| sleep_hrs | 1.03 | 2 | 1.01 | -- | sleep_hrs | 1.03 | 2 | 1.01 | -- |

# **Table S17:** collinearity assessment for porn addiction scale

| **PPCS** | | | | |
| --- | --- | --- | --- | --- |
|  |  |  |  |  |
| **predictor** | **GVIF** | **Df** | **GVIF^(1/(2*Df))** | **Interacts With** |
| gad7_score | 2.13 | 1 | 1.46 | -- |
| phq9_score | 2.13 | 1 | 1.46 | -- |
| pss_score | 1.55 | 3 | 1.08 | gender |
| gender | 1.55 | 3 | 1.08 | pss_score |
| age | 1.27 | 1 | 1.13 | -- |
| study_country | 1.36 | 2 | 1.08 | -- |
| income | 1.05 | 2 | 1.01 | -- |
| sleep_hrs | 1.03 | 2 | 1.01 | -- |

# **Table S18:** fit indices of the SEM mediation models

| **Model** | **χ² (df)** | **χ²/df** | **p** | **Bollen-Stine p** | **CFI** | **TLI** | **SRMR** | **RMSEA [90% CI]** |
| --- | --- | --- | --- | --- | --- | --- | --- | --- |
| 1. Stress → IAT | 17.23 (8) | 2.15 | 0.028 | > .99 | 0.994 | 0.978 | 0.014 | .030 [.009, .050] |
| 2. Stress → PPCS | 17.23 (8) | 2.15 | 0.028 | > .99 | 0.994 | 0.978 | 0.015 | .030 [.009, .050] |
| 3. Sleep → IAT | 12.07 (4) | 3.02 | 0.017 | > .99 | 0.995 | 0.962 | 0.012 | .040 [.015, .066] |
| 4. Sleep → PPCS | 12.07 (4) | 3.02 | 0.017 | > .99 | 0.995 | 0.962 | 0.013 | .040 [.015, .066] |

# **Figure S19:** Full parameter estimates for the parallel dual-mediator structural equation model with pornography addiction (PPCS score) as the outcome: paths through depression (PHQ-9) and anxiety (GAD-7), with perceived stress (PSS) as the exposure. Bootstrap standard errors based on 5,000 resamples (N = 1,284).

P-value (Bollen-Stine bootstrap) = > 0.99

| term | op | label | estimate | std.error | statistic | p.value | conf.low | conf.high | std.lv | std.all | std.nox |
| --- | --- | --- | --- | --- | --- | --- | --- | --- | --- | --- | --- |
| phq9_score ~ pss_score | Regression~ | a1 | 0.69 | 0.03 | 21.40 | <0.0001 | 0.63 | 0.76 | 0.69 | 0.51 | 0.11 |
| phq9_score ~ gender | Regression~ |  | 0.49 | 0.33 | 1.51 | 0.1299 | -0.13 | 1.15 | 0.49 | 0.04 | 0.07 |
| phq9_score ~ age | Regression~ |  | -0.11 | 0.07 | -1.43 | 0.152 | -0.25 | 0.04 | -0.11 | -0.04 | -0.02 |
| phq9_score ~ study_countryLiyba | Regression~ |  | -0.84 | 0.49 | -1.74 | 0.0827 | -1.76 | 0.12 | -0.84 | -0.05 | -0.13 |
| phq9_score ~ study_countrySudan | Regression~ |  | -1.07 | 0.37 | -2.93 | 0.0034 | -1.80 | -0.36 | -1.07 | -0.08 | -0.16 |
| gad7_score ~ pss_score | Regression~ | a2 | 0.58 | 0.03 | 20.87 | <0.0001 | 0.52 | 0.63 | 0.58 | 0.50 | 0.10 |
| gad7_score ~ gender | Regression~ |  | 0.08 | 0.27 | 0.29 | 0.7728 | -0.44 | 0.62 | 0.08 | 0.01 | 0.01 |
| gad7_score ~ age | Regression~ |  | 0.03 | 0.06 | 0.48 | 0.6312 | -0.09 | 0.15 | 0.03 | 0.01 | 0.01 |
| gad7_score ~ study_countryLiyba | Regression~ |  | -0.82 | 0.45 | -1.83 | 0.0677 | -1.68 | 0.08 | -0.82 | -0.05 | -0.15 |
| gad7_score ~ study_countrySudan | Regression~ |  | -1.41 | 0.30 | -4.71 | <0.0001 | -1.99 | -0.82 | -1.41 | -0.12 | -0.25 |
| ppcs_score ~ pss_score | Regression~ | c_prime | -0.20 | 0.15 | -1.29 | 0.1959 | -0.51 | 0.10 | -0.20 | -0.04 | -0.01 |
| ppcs_score ~ phq9_score | Regression~ | b1 | 0.79 | 0.15 | 5.15 | <0.0001 | 0.50 | 1.11 | 0.79 | 0.23 | 0.23 |
| ppcs_score ~ gad7_score | Regression~ | b2 | 0.54 | 0.17 | 3.23 | 0.0013 | 0.20 | 0.86 | 0.54 | 0.13 | 0.13 |
| ppcs_score ~ gender | Regression~ |  | 10.43 | 1.29 | 8.09 | <0.0001 | 7.91 | 12.90 | 10.43 | 0.22 | 0.46 |
| ppcs_score ~ age | Regression~ |  | -0.37 | 0.27 | -1.36 | 0.1744 | -0.90 | 0.18 | -0.37 | -0.04 | -0.02 |
| ppcs_score ~ study_countryLiyba | Regression~ |  | 0.29 | 1.75 | 0.17 | 0.8688 | -3.20 | 3.74 | 0.29 | 0.00 | 0.01 |
| ppcs_score ~ study_countrySudan | Regression~ |  | 1.31 | 1.34 | 0.98 | 0.3285 | -1.29 | 3.99 | 1.31 | 0.03 | 0.06 |
| ppcs_score ~ incomeNot_enough | Regression~ |  | 0.14 | 2.20 | 0.06 | 0.9487 | -4.07 | 4.58 | 0.14 | 0.00 | 0.01 |
| ppcs_score ~ incomeEnough_and_saving | Regression~ |  | 0.20 | 1.26 | 0.16 | 0.8717 | -2.21 | 2.67 | 0.20 | 0.00 | 0.01 |
| ppcs_score ~ sleep_hrsLess_than_6_hours | Regression~ |  | -0.83 | 1.26 | -0.66 | 0.5104 | -3.31 | 1.63 | -0.83 | -0.02 | -0.04 |
| ppcs_score ~ sleep_hrsMore_than_9_hours | Regression~ |  | 7.52 | 3.26 | 2.31 | 0.0211 | 1.28 | 14.16 | 7.52 | 0.07 | 0.33 |
| phq9_score ~~ gad7_score | Covariances: |  | 15.28 | 0.89 | 17.13 | <0.0001 | 13.48 | 17.01 | 15.28 | 0.58 | 0.58 |
| phq9_score ~~ phq9_score | Variances: |  | 31.08 | 1.36 | 22.90 | <0.0001 | 28.37 | 33.65 | 31.08 | 0.72 | 0.72 |
| gad7_score ~~ gad7_score | Variances: |  | 22.04 | 0.83 | 26.55 | <0.0001 | 20.32 | 23.60 | 22.04 | 0.72 | 0.72 |
| ppcs_score ~~ ppcs_score | Variances: |  | 432.89 | 24.74 | 17.50 | <0.0001 | 380.85 | 478.41 | 432.89 | 0.85 | 0.85 |
| ind_phq9 := a1*b1 | Defined Parameters: | ind_phq9 | 0.55 | 0.11 | 4.96 | <0.0001 | 0.34 | 0.78 | 0.55 | 0.12 | 0.02 |
| ind_gad7 := a2*b2 | Defined Parameters: | ind_gad7 | 0.31 | 0.10 | 3.16 | 0.0016 | 0.12 | 0.50 | 0.31 | 0.07 | 0.01 |
| ind_total := ind_phq9+ind_gad7 | Defined Parameters: | ind_total | 0.86 | 0.11 | 8.06 | <0.0001 | 0.66 | 1.08 | 0.86 | 0.18 | 0.04 |
| total := c_prime+ind_total | Defined Parameters: | total | 0.66 | 0.14 | 4.79 | <0.0001 | 0.40 | 0.94 | 0.66 | 0.14 | 0.03 |

| index | value |
| --- | --- |
| npar | 25 |
| fmin | 0.006709759 |
| chisq | 17.23066044 |
| df | 8 |
| pvalue | 0.02779487 |
| baseline.chisq | 1622.403591 |
| baseline.df | 30 |
| baseline.pvalue | 0 |
| cfi | 0.994 |
| tli | 0.978 |
| nnfi | 0.978 |
| rfi | 0.960 |
| nfi | 0.989 |
| pnfi | 0.264 |
| ifi | 0.994 |
| rni | 0.994 |
| logl | -13287.1 |
| unrestricted.logl | -13278.5 |
| aic | 26624.2 |
| bic | 26753.2 |
| ntotal | 1284 |
| bic2 | 26673.7 |
| rmsea | 0.030 |
| rmsea.ci.lower | 0.009 |
| rmsea.ci.upper | 0.050 |
| rmsea.ci.level | 0.90 |
| rmsea.pvalue | 0.95398197 |
| rmsea.close.h0 | 0.05 |
| rmsea.notclose.pvalue | 2.64E-06 |
| rmsea.notclose.h0 | 0.08 |
| rmr | 0.057 |
| rmr_nomean | 0.057 |
| srmr | 0.015 |
| srmr_bentler | 0.015 |
| srmr_bentler_nomean | 0.015 |
| crmr | 0.016 |
| crmr_nomean | 0.016 |
| srmr_mplus | 0.015 |
| srmr_mplus_nomean | 0.015 |
| cn_05 | 1156.6 |
| cn_01 | 1498.1 |
| gfi | 0.992 |
| agfi | 0.921 |
| pgfi | 0.102 |
| mfi | 0.996 |
| ecvi | 0.052 |

# **Table S20.** Full parameter estimates for the parallel dual-mediator structural equation model with internet addiction (IAT score) as the outcome: paths through depression (PHQ-9) and anxiety (GAD-7), with perceived stress (PSS) as the exposure. Bootstrap standard errors based on 5,000 resamples (N = 1,284).

P-value (Bollen-Stine bootstrap) = > 0.99

| term | op | label | estimate | std.error | statistic | p.value | conf.low | conf.high | std.lv | std.all | std.nox |
| --- | --- | --- | --- | --- | --- | --- | --- | --- | --- | --- | --- |
| phq9_score ~ pss_score | Regression~ | a1 | 0.69 | 0.03 | 21.14 | <0.0001 | 0.631 | 0.759 | 0.693 | 0.508 | 0.105 |
| phq9_score ~ gender | Regression~ |  | 0.49 | 0.33 | 1.48 | 0.14 | -0.169 | 1.131 | 0.493 | 0.036 | 0.075 |
| phq9_score ~ age | Regression~ |  | -0.11 | 0.07 | -1.43 | 0.1537 | -0.251 | 0.037 | -0.106 | -0.038 | -0.016 |
| phq9_score ~ study_countryLiyba | Regression~ |  | -0.84 | 0.48 | -1.74 | 0.0815 | -1.782 | 0.090 | -0.844 | -0.045 | -0.128 |
| phq9_score ~ study_countrySudan | Regression~ |  | -1.07 | 0.37 | -2.94 | 0.0033 | -1.779 | -0.348 | -1.075 | -0.079 | -0.163 |
| gad7_score ~ pss_score | Regression~ | a2 | 0.58 | 0.03 | 20.86 | <0.0001 | 0.524 | 0.632 | 0.578 | 0.502 | 0.104 |
| gad7_score ~ gender | Regression~ |  | 0.08 | 0.27 | 0.29 | 0.7746 | -0.446 | 0.616 | 0.078 | 0.007 | 0.014 |
| gad7_score ~ age | Regression~ |  | 0.03 | 0.06 | 0.48 | 0.6317 | -0.095 | 0.153 | 0.030 | 0.013 | 0.005 |
| gad7_score ~ study_countryLiyba | Regression~ |  | -0.82 | 0.44 | -1.85 | 0.0641 | -1.689 | 0.033 | -0.816 | -0.052 | -0.147 |
| gad7_score ~ study_countrySudan | Regression~ |  | -1.41 | 0.30 | -4.69 | <0.0001 | -1.985 | -0.811 | -1.413 | -0.123 | -0.254 |
| iat_score ~ pss_score | Regression~ | c_prime | 0.43 | 0.13 | 3.34 | 0.0008 | 0.172 | 0.680 | 0.429 | 0.106 | 0.022 |
| iat_score ~ phq9_score | Regression~ | b1 | 0.53 | 0.12 | 4.28 | <0.0001 | 0.287 | 0.773 | 0.525 | 0.176 | 0.176 |
| iat_score ~ gad7_score | Regression~ | b2 | 0.41 | 0.14 | 2.95 | 0.0032 | 0.129 | 0.674 | 0.408 | 0.116 | 0.116 |
| iat_score ~ gender | Regression~ |  | 0.37 | 1.06 | 0.35 | 0.7283 | -1.749 | 2.474 | 0.368 | 0.009 | 0.019 |
| iat_score ~ age | Regression~ |  | -0.29 | 0.24 | -1.19 | 0.2322 | -0.762 | 0.190 | -0.288 | -0.035 | -0.015 |
| iat_score ~ study_countryLiyba | Regression~ |  | -5.81 | 1.65 | -3.52 | 0.0004 | -9.007 | -2.515 | -5.807 | -0.104 | -0.296 |
| iat_score ~ study_countrySudan | Regression~ |  | -2.60 | 1.21 | -2.15 | 0.0319 | -5.016 | -0.213 | -2.600 | -0.064 | -0.133 |
| iat_score ~ incomeNot_enough | Regression~ |  | -3.02 | 2.03 | -1.49 | 0.1362 | -7.020 | 0.927 | -3.020 | -0.043 | -0.154 |
| iat_score ~ incomeEnough_and_saving | Regression~ |  | 0.77 | 1.07 | 0.72 | 0.4728 | -1.330 | 2.891 | 0.767 | 0.019 | 0.039 |
| iat_score ~ sleep_hrsLess_than_6_hours | Regression~ |  | -2.75 | 1.11 | -2.48 | 0.0131 | -4.905 | -0.577 | -2.747 | -0.065 | -0.140 |
| iat_score ~ sleep_hrsMore_than_9_hours | Regression~ |  | 0.97 | 2.95 | 0.33 | 0.7435 | -4.621 | 6.774 | 0.966 | 0.010 | 0.049 |
| phq9_score ~~ gad7_score | Covariances: |  | 15.28 | 0.91 | 16.83 | <0.0001 | 13.497 | 17.017 | 15.280 | 0.584 | 0.584 |
| phq9_score ~~ phq9_score | Variances: |  | 31.08 | 1.37 | 22.70 | <0.0001 | 28.366 | 33.639 | 31.076 | 0.716 | 0.716 |
| gad7_score ~~ gad7_score | Variances: |  | 22.04 | 0.83 | 26.69 | <0.0001 | 20.363 | 23.582 | 22.038 | 0.715 | 0.715 |
| iat_score ~~ iat_score | Variances: |  | 325.92 | 12.28 | 26.53 | <0.0001 | 299.456 | 346.755 | 325.922 | 0.847 | 0.847 |
| ind_phq9 := a1*b1 | Defined Parameters: | ind_phq9 | 0.36 | 0.09 | 4.19 | <0.0001 | 0.198 | 0.542 | 0.364 | 0.090 | 0.019 |
| ind_gad7 := a2*b2 | Defined Parameters: | ind_gad7 | 0.24 | 0.08 | 2.91 | 0.0037 | 0.074 | 0.396 | 0.236 | 0.058 | 0.012 |
| ind_total := ind_phq9+ind_gad7 | Defined Parameters: | ind_total | 0.60 | 0.08 | 7.15 | <0.0001 | 0.440 | 0.770 | 0.600 | 0.148 | 0.031 |
| total := c_prime+ind_total | Defined Parameters: | total | 1.03 | 0.11 | 9.32 | <0.0001 | 0.815 | 1.247 | 1.029 | 0.253 | 0.052 |

| index | value |
| --- | --- |
| npar | 25 |
| fmin | 0.00671 |
| chisq | 17.23066 |
| df | 8 |
| pvalue | 0.027795 |
| baseline.chisq | 1620.505 |
| baseline.df | 30 |
| baseline.pvalue | 0 |
| cfi | 0.994196 |
| tli | 0.978236 |
| nnfi | 0.978236 |
| rfi | 0.960127 |
| nfi | 0.989367 |
| pnfi | 0.263831 |
| ifi | 0.994276 |
| rni | 0.994196 |
| logl | -13104.9 |
| unrestricted.logl | -13096.3 |
| aic | 26259.77 |
| bic | 26388.71 |
| ntotal | 1284 |
| bic2 | 26309.3 |
| rmsea | 0.029977 |
| rmsea.ci.lower | 0.00944 |
| rmsea.ci.upper | 0.049573 |
| rmsea.ci.level | 0.9 |
| rmsea.pvalue | 0.953982 |
| rmsea.close.h0 | 0.05 |
| rmsea.notclose.pvalue | 2.64E-06 |
| rmsea.notclose.h0 | 0.08 |
| rmr | 0.174173 |
| rmr_nomean | 0.174173 |
| srmr | 0.014455 |
| srmr_bentler | 0.014455 |
| srmr_bentler_nomean | 0.014455 |
| crmr | 0.015703 |
| crmr_nomean | 0.015703 |
| srmr_mplus | 0.014449 |
| srmr_mplus_nomean | 0.014449 |
| cn_05 | 1156.579 |
| cn_01 | 1498.091 |
| gfi | 0.991905 |
| agfi | 0.921071 |
| pgfi | 0.101734 |
| mfi | 0.996412 |
| ecvi | 0.05236 |

# **Table S21.** Full parameter estimates for the parallel dual-mediator structural equation model with pornography addiction (PPCS score) as the outcome: paths through depression (PHQ-9) and anxiety (GAD-7), with sleep duration as the exposure and perceived stress (PSS) as a covariate. Bootstrap standard errors based on 5,000 resamples (N = 1,284).

P-value (Bollen-Stine bootstrap) = > 0.99

| term | op | label | estimate | std.error | statistic | p.value | conf.low | conf.high | std.lv | std.all | std.nox |
| --- | --- | --- | --- | --- | --- | --- | --- | --- | --- | --- | --- |
| phq9_score ~ sleep_hrsLess_than_6_hours | Regression~ | a1_less | 0.33 | 0.35 | 0.95 | 0.3409 | -0.35 | 1.01 | 0.33 | 0.02 | 0.05 |
| phq9_score ~ sleep_hrsMore_than_9_hours | Regression~ | a1_more | 0.83 | 0.77 | 1.07 | 0.2841 | -0.65 | 2.38 | 0.83 | 0.03 | 0.13 |
| phq9_score ~ pss_score | Regression~ |  | 0.69 | 0.03 | 21.18 | <0.0001 | 0.63 | 0.76 | 0.69 | 0.51 | 0.11 |
| phq9_score ~ gender | Regression~ |  | 0.50 | 0.33 | 1.50 | 0.1334 | -0.15 | 1.14 | 0.50 | 0.04 | 0.08 |
| phq9_score ~ age | Regression~ |  | -0.11 | 0.07 | -1.44 | 0.1507 | -0.25 | 0.04 | -0.11 | -0.04 | -0.02 |
| phq9_score ~ study_countryLiyba | Regression~ |  | -0.81 | 0.48 | -1.68 | 0.0933 | -1.76 | 0.14 | -0.81 | -0.04 | -0.12 |
| phq9_score ~ study_countrySudan | Regression~ |  | -1.04 | 0.37 | -2.85 | 0.0043 | -1.75 | -0.32 | -1.04 | -0.08 | -0.16 |
| gad7_score ~ sleep_hrsLess_than_6_hours | Regression~ | a2_less | 0.58 | 0.29 | 1.97 | 0.0483 | 0.02 | 1.17 | 0.58 | 0.05 | 0.10 |
| gad7_score ~ sleep_hrsMore_than_9_hours | Regression~ | a2_more | 0.41 | 0.65 | 0.64 | 0.5233 | -0.86 | 1.73 | 0.41 | 0.02 | 0.07 |
| gad7_score ~ pss_score | Regression~ |  | 0.58 | 0.03 | 20.69 | <0.0001 | 0.52 | 0.63 | 0.58 | 0.50 | 0.10 |
| gad7_score ~ gender | Regression~ |  | 0.07 | 0.27 | 0.24 | 0.8116 | -0.46 | 0.61 | 0.07 | 0.01 | 0.01 |
| gad7_score ~ age | Regression~ |  | 0.03 | 0.06 | 0.53 | 0.5969 | -0.09 | 0.16 | 0.03 | 0.01 | 0.01 |
| gad7_score ~ study_countryLiyba | Regression~ |  | -0.82 | 0.44 | -1.84 | 0.0656 | -1.69 | 0.04 | -0.82 | -0.05 | -0.15 |
| gad7_score ~ study_countrySudan | Regression~ |  | -1.38 | 0.30 | -4.56 | <0.0001 | -1.95 | -0.77 | -1.38 | -0.12 | -0.25 |
| ppcs_score ~ phq9_score | Regression~ | b1 | 0.79 | 0.15 | 5.18 | <0.0001 | 0.50 | 1.10 | 0.79 | 0.23 | 0.23 |
| ppcs_score ~ gad7_score | Regression~ | b2 | 0.54 | 0.17 | 3.17 | 0.0015 | 0.21 | 0.87 | 0.54 | 0.13 | 0.13 |
| ppcs_score ~ sleep_hrsLess_than_6_hours | Regression~ | c_prime_less | -0.83 | 1.27 | -0.65 | 0.514 | -3.28 | 1.72 | -0.83 | -0.02 | -0.04 |
| ppcs_score ~ sleep_hrsMore_than_9_hours | Regression~ | c_prime_more | 7.52 | 3.18 | 2.37 | 0.018 | 1.48 | 13.84 | 7.52 | 0.07 | 0.33 |
| ppcs_score ~ pss_score | Regression~ |  | -0.20 | 0.16 | -1.29 | 0.1986 | -0.50 | 0.11 | -0.20 | -0.04 | -0.01 |
| ppcs_score ~ gender | Regression~ |  | 10.43 | 1.30 | 8.00 | <0.0001 | 7.75 | 12.96 | 10.43 | 0.22 | 0.46 |
| ppcs_score ~ age | Regression~ |  | -0.37 | 0.27 | -1.37 | 0.1717 | -0.91 | 0.17 | -0.37 | -0.04 | -0.02 |
| ppcs_score ~ study_countryLiyba | Regression~ |  | 0.29 | 1.71 | 0.17 | 0.8658 | -3.05 | 3.69 | 0.29 | 0.00 | 0.01 |
| ppcs_score ~ study_countrySudan | Regression~ |  | 1.31 | 1.35 | 0.97 | 0.3325 | -1.30 | 4.01 | 1.31 | 0.03 | 0.06 |
| ppcs_score ~ incomeNot_enough | Regression~ |  | 0.14 | 2.23 | 0.06 | 0.9494 | -4.30 | 4.46 | 0.14 | 0.00 | 0.01 |
| ppcs_score ~ incomeEnough_and_saving | Regression~ |  | 0.20 | 1.24 | 0.16 | 0.8694 | -2.20 | 2.61 | 0.20 | 0.00 | 0.01 |
| phq9_score ~~ gad7_score | Covariances: |  | 15.23 | 0.91 | 16.74 | <0.0001 | 13.44 | 16.95 | 15.23 | 0.58 | 0.58 |
| phq9_score ~~ phq9_score | Variances: |  | 31.03 | 1.37 | 22.72 | <0.0001 | 28.28 | 33.53 | 31.03 | 0.72 | 0.72 |
| gad7_score ~~ gad7_score | Variances: |  | 21.97 | 0.82 | 26.66 | <0.0001 | 20.25 | 23.46 | 21.97 | 0.71 | 0.71 |
| ppcs_score ~~ ppcs_score | Variances: |  | 432.89 | 24.65 | 17.56 | <0.0001 | 381.83 | 477.64 | 432.89 | 0.85 | 0.85 |
| ind_less_phq9 := a1_less*b1 | Defined Parameters: | ind_less_phq9 | 0.26 | 0.28 | 0.94 | 0.3475 | -0.29 | 0.82 | 0.26 | 0.01 | 0.01 |
| ind_less_gad7 := a2_less*b2 | Defined Parameters: | ind_less_gad7 | 0.31 | 0.20 | 1.57 | 0.1168 | 0.01 | 0.77 | 0.31 | 0.01 | 0.01 |
| ind_less_total := ind_less_phq9+ind_less_gad7 | Defined Parameters: | ind_less_total | 0.58 | 0.41 | 1.42 | 0.1565 | -0.21 | 1.41 | 0.58 | 0.01 | 0.03 |
| ind_more_phq9 := a1_more*b1 | Defined Parameters: | ind_more_phq9 | 0.65 | 0.64 | 1.03 | 0.3045 | -0.50 | 2.01 | 0.65 | 0.01 | 0.03 |
| ind_more_gad7 := a2_more*b2 | Defined Parameters: | ind_more_gad7 | 0.22 | 0.38 | 0.58 | 0.5598 | -0.47 | 1.08 | 0.22 | 0.00 | 0.01 |
| ind_more_total := ind_more_phq9+ind_more_gad7 | Defined Parameters: | ind_more_total | 0.88 | 0.87 | 1.00 | 0.3152 | -0.77 | 2.65 | 0.88 | 0.01 | 0.04 |
| total_less := c_prime_less+ind_less_total | Defined Parameters: | total_less | -0.25 | 1.33 | -0.19 | 0.8485 | -2.77 | 2.36 | -0.25 | -0.01 | -0.01 |
| total_more := c_prime_more+ind_more_total | Defined Parameters: | total_more | 8.39 | 3.51 | 2.39 | 0.0167 | 1.75 | 15.41 | 8.39 | 0.08 | 0.37 |

| index | value |
| --- | --- |
| npar | 29 |
| fmin | 0.004702 |
| chisq | 12.07407 |
| df | 4 |
| pvalue | 0.016809 |
| baseline.chisq | 1622.404 |
| baseline.df | 30 |
| baseline.pvalue | 0 |
| cfi | 0.99493 |
| tli | 0.961972 |
| nnfi | 0.961972 |
| rfi | 0.944184 |
| nfi | 0.992558 |
| pnfi | 0.132341 |
| ifi | 0.995011 |
| rni | 0.99493 |
| logl | -13284.5 |
| unrestricted.logl | -13278.5 |
| aic | 26627.06 |
| bic | 26776.63 |
| ntotal | 1284 |
| bic2 | 26684.51 |
| rmsea | 0.039649 |
| rmsea.ci.lower | 0.01514 |
| rmsea.ci.upper | 0.066247 |
| rmsea.ci.level | 0.9 |
| rmsea.pvalue | 0.705178 |
| rmsea.close.h0 | 0.05 |
| rmsea.notclose.pvalue | 0.004738 |
| rmsea.notclose.h0 | 0.08 |
| rmr | 0.040988 |
| rmr_nomean | 0.040988 |
| srmr | 0.012526 |
| srmr_bentler | 0.012526 |
| srmr_bentler_nomean | 0.012526 |
| crmr | 0.013618 |
| crmr_nomean | 0.013618 |
| srmr_mplus | 0.012526 |
| srmr_mplus_nomean | 0.012526 |
| cn_05 | 1009.959 |
| cn_01 | 1412.892 |
| gfi | 0.994291 |
| agfi | 0.888665 |
| pgfi | 0.050989 |
| mfi | 0.996861 |
| ecvi | 0.054575 |

# **Table S22.** Full parameter estimates for the parallel dual-mediator structural equation model with internet addiction (IAT score) as the outcome: paths through depression (PHQ-9) and anxiety (GAD-7), with sleep duration as the exposure and perceived stress (PSS) as a covariate. Bootstrap standard errors based on 5,000 resamples (N = 1,284).

P-value (Bollen-Stine bootstrap) = > 0.99

| term | op | label | estimate | std.error | statistic | p.value | conf.low | conf.high | std.lv | std.all | std.nox |
| --- | --- | --- | --- | --- | --- | --- | --- | --- | --- | --- | --- |
| phq9_score ~ sleep_hrsLess_than_6_hours | Regression~ | a1_less | 0.333638 | 0.350284 | 0.952479 | 0.3409 | -0.34748 | 1.005143 | 0.333638 | 0.023425 | 0.050651 |
| phq9_score ~ sleep_hrsMore_than_9_hours | Regression~ | a1_more | 0.826669 | 0.771729 | 1.07119 | 0.2841 | -0.64801 | 2.375989 | 0.826669 | 0.026277 | 0.1255 |
| phq9_score ~ pss_score | Regression~ |  | 0.692392 | 0.032694 | 21.17802 | <0.0001 | 0.629592 | 0.758225 | 0.692392 | 0.507377 | 0.105115 |
| phq9_score ~ gender | Regression~ |  | 0.502706 | 0.33493 | 1.500928 | 0.1334 | -0.15119 | 1.143794 | 0.502706 | 0.037059 | 0.076318 |
| phq9_score ~ age | Regression~ |  | -0.10735 | 0.074697 | -1.43717 | 0.1507 | -0.25346 | 0.036283 | -0.10735 | -0.03885 | -0.0163 |
| phq9_score ~ study_countryLiyba | Regression~ |  | -0.81219 | 0.484012 | -1.67804 | 0.0933 | -1.75895 | 0.136984 | -0.81219 | -0.04349 | -0.1233 |
| phq9_score ~ study_countrySudan | Regression~ |  | -1.04267 | 0.365228 | -2.85483 | 0.0043 | -1.74585 | -0.31815 | -1.04267 | -0.07662 | -0.15829 |
| gad7_score ~ sleep_hrsLess_than_6_hours | Regression~ | a2_less | 0.581888 | 0.29469 | 1.974578 | 0.0483 | 0.022917 | 1.171638 | 0.581888 | 0.048475 | 0.104816 |
| gad7_score ~ sleep_hrsMore_than_9_hours | Regression~ | a2_more | 0.413804 | 0.648296 | 0.638296 | 0.5233 | -0.85875 | 1.725277 | 0.413804 | 0.015607 | 0.074539 |
| gad7_score ~ pss_score | Regression~ |  | 0.576273 | 0.027851 | 20.69156 | <0.0001 | 0.522377 | 0.630917 | 0.576273 | 0.501053 | 0.103805 |
| gad7_score ~ gender | Regression~ |  | 0.065199 | 0.273564 | 0.238331 | 0.8116 | -0.45731 | 0.605987 | 0.065199 | 0.005703 | 0.011744 |
| gad7_score ~ age | Regression~ |  | 0.033122 | 0.062635 | 0.528805 | 0.5969 | -0.09094 | 0.155263 | 0.033122 | 0.014222 | 0.005966 |
| gad7_score ~ study_countryLiyba | Regression~ |  | -0.81687 | 0.44366 | -1.84121 | 0.0656 | -1.68725 | 0.038632 | -0.81687 | -0.0519 | -0.14714 |
| gad7_score ~ study_countrySudan | Regression~ |  | -1.37574 | 0.301886 | -4.55715 | <0.0001 | -1.95274 | -0.77282 | -1.37574 | -0.11995 | -0.24781 |
| iat_score ~ phq9_score | Regression~ | b1 | 0.525176 | 0.122611 | 4.283276 | <0.0001 | 0.28741 | 0.773217 | 0.525176 | 0.176479 | 0.176479 |
| iat_score ~ gad7_score | Regression~ | b2 | 0.408247 | 0.1384 | 2.949757 | 0.0032 | 0.129282 | 0.673868 | 0.408247 | 0.11562 | 0.11562 |
| iat_score ~ sleep_hrsLess_than_6_hours | Regression~ | c_prime_less | -2.74738 | 1.106871 | -2.48212 | 0.0131 | -4.90524 | -0.57685 | -2.74738 | -0.06482 | -0.14016 |
| iat_score ~ sleep_hrsMore_than_9_hours | Regression~ | c_prime_more | 0.965941 | 2.951952 | 0.327221 | 0.7435 | -4.62118 | 6.773653 | 0.965941 | 0.010318 | 0.049278 |
| iat_score ~ pss_score | Regression~ |  | 0.429202 | 0.128417 | 3.342256 | 0.0008 | 0.171957 | 0.680368 | 0.429202 | 0.105689 | 0.021896 |
| iat_score ~ gender | Regression~ |  | 0.368441 | 1.060543 | 0.347408 | 0.7283 | -1.74875 | 2.473821 | 0.368441 | 0.009127 | 0.018796 |
| iat_score ~ age | Regression~ |  | -0.28783 | 0.240943 | -1.19462 | 0.2322 | -0.76235 | 0.189603 | -0.28783 | -0.035 | -0.01468 |
| iat_score ~ study_countryLiyba | Regression~ |  | -5.80652 | 1.648619 | -3.52205 | 0.0004 | -9.00694 | -2.51547 | -5.80652 | -0.10449 | -0.29622 |
| iat_score ~ study_countrySudan | Regression~ |  | -2.60012 | 1.211856 | -2.14557 | 0.0319 | -5.01614 | -0.21283 | -2.60012 | -0.0642 | -0.13265 |
| iat_score ~ incomeNot_enough | Regression~ |  | -3.02024 | 2.026823 | -1.49014 | 0.1362 | -7.01978 | 0.926815 | -3.02024 | -0.04294 | -0.15408 |
| iat_score ~ incomeEnough_and_saving | Regression~ |  | 0.767173 | 1.068669 | 0.717877 | 0.4728 | -1.32949 | 2.891254 | 0.767173 | 0.019388 | 0.039137 |
| phq9_score ~~ gad7_score | Covariances: |  | 15.23215 | 0.910096 | 16.73685 | <0.0001 | 13.44381 | 16.94569 | 15.23215 | 0.583446 | 0.583446 |
| phq9_score ~~ phq9_score | Variances: |  | 31.03007 | 1.365887 | 22.71789 | <0.0001 | 28.27698 | 33.5336 | 31.03007 | 0.715165 | 0.715165 |
| gad7_score ~~ gad7_score | Variances: |  | 21.96535 | 0.823945 | 26.65875 | <0.0001 | 20.25057 | 23.46427 | 21.96535 | 0.712715 | 0.712715 |
| iat_score ~~ iat_score | Variances: |  | 325.9219 | 12.28377 | 26.53273 | <0.0001 | 299.4563 | 346.7554 | 325.9219 | 0.848228 | 0.848228 |
| ind_less_phq9 := a1_less*b1 | Defined Parameters: | ind_less_phq9 | 0.175219 | 0.192369 | 0.910848 | 0.3624 | -0.19497 | 0.584241 | 0.175219 | 0.004134 | 0.008939 |
| ind_less_gad7 := a2_less*b2 | Defined Parameters: | ind_less_gad7 | 0.237554 | 0.150032 | 1.583358 | 0.1133 | 0.003129 | 0.568495 | 0.237554 | 0.005605 | 0.012119 |
| ind_less_total := ind_less_phq9+ind_less_gad7 | Defined Parameters: | ind_less_total | 0.412773 | 0.285935 | 1.443589 | 0.1489 | -0.13715 | 1.004991 | 0.412773 | 0.009739 | 0.021058 |
| ind_more_phq9 := a1_more*b1 | Defined Parameters: | ind_more_phq9 | 0.434147 | 0.425584 | 1.02012 | 0.3077 | -0.34818 | 1.329653 | 0.434147 | 0.004637 | 0.022148 |
| ind_more_gad7 := a2_more*b2 | Defined Parameters: | ind_more_gad7 | 0.168934 | 0.286306 | 0.590048 | 0.5552 | -0.37011 | 0.805216 | 0.168934 | 0.001804 | 0.008618 |
| ind_more_total := ind_more_phq9+ind_more_gad7 | Defined Parameters: | ind_more_total | 0.603081 | 0.606774 | 0.993914 | 0.3203 | -0.57744 | 1.837072 | 0.603081 | 0.006442 | 0.030766 |
| total_less := c_prime_less+ind_less_total | Defined Parameters: | total_less | -2.33461 | 1.138319 | -2.05093 | 0.0403 | -4.56658 | -0.09283 | -2.33461 | -0.05508 | -0.1191 |
| total_more := c_prime_more+ind_more_total | Defined Parameters: | total_more | 1.569022 | 3.022479 | 0.519118 | 0.6037 | -4.22563 | 7.628475 | 1.569022 | 0.016759 | 0.080044 |

| index | value |
| --- | --- |
| npar | 29 |
| fmin | 0.004701742 |
| chisq | 12.07407405 |
| df | 4 |
| pvalue | 0.016808849 |
| baseline.chisq | 1620.505458 |
| baseline.df | 30 |
| baseline.pvalue | 0 |
| cfi | 0.99492358 |
| tli | 0.961926848 |
| nnfi | 0.961926848 |
| rfi | 0.944118945 |
| nfi | 0.992549193 |
| pnfi | 0.132339892 |
| ifi | 0.995005229 |
| rni | 0.99492358 |
| logl | -13102.3056 |
| unrestricted.logl | -13096.26856 |
| aic | 26262.6112 |
| bic | 26412.18553 |
| ntotal | 1284 |
| bic2 | 26320.06711 |
| rmsea | 0.039649148 |
| rmsea.ci.lower | 0.01513997 |
| rmsea.ci.upper | 0.066247124 |
| rmsea.ci.level | 0.9 |
| rmsea.pvalue | 0.705178324 |
| rmsea.close.h0 | 0.05 |
| rmsea.notclose.pvalue | 0.004738166 |
| rmsea.notclose.h0 | 0.08 |
| rmr | 0.113745381 |
| rmr_nomean | 0.113745381 |
| srmr | 0.01239838 |
| srmr_bentler | 0.01239838 |
| srmr_bentler_nomean | 0.01239838 |
| crmr | 0.013472758 |
| crmr_nomean | 0.013472758 |
| srmr_mplus | 0.012395179 |
| srmr_mplus_nomean | 0.012395179 |
| cn_05 | 1009.958868 |
| cn_01 | 1412.891963 |
| gfi | 0.994314071 |
| agfi | 0.889124393 |
| pgfi | 0.050990465 |
| mfi | 0.996860828 |
| ecvi | 0.054574824 |

# **Figure S1:** Histogram and Q-Q plot for age with normality test results


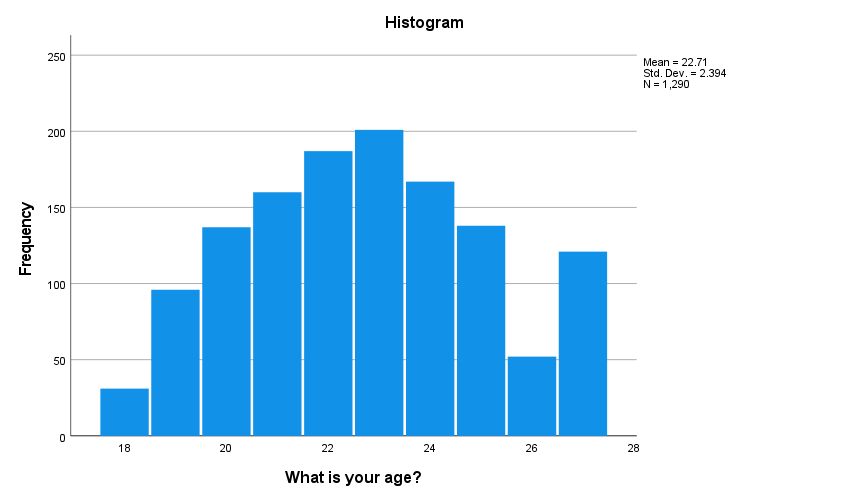


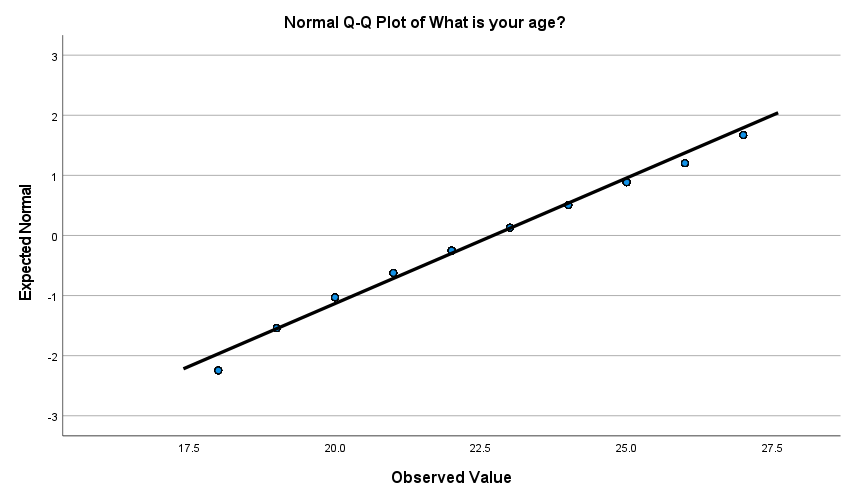


| **Tests of Normality** | | | | | | |
| --- | --- | --- | --- | --- | --- | --- |
|  | Kolmogorov-Smirnov^a^ | | | Shapiro-Wilk | | |
|  | Statistic | df | P value | Statistic | df | P value |
| What is your age? | .091 | 1290 | <0.001 | .962 | 1290 | <0.001 |
| a. Lilliefors Significance Correction | | | | | | |

# **Figure S2**: IAT individual items Likert plot


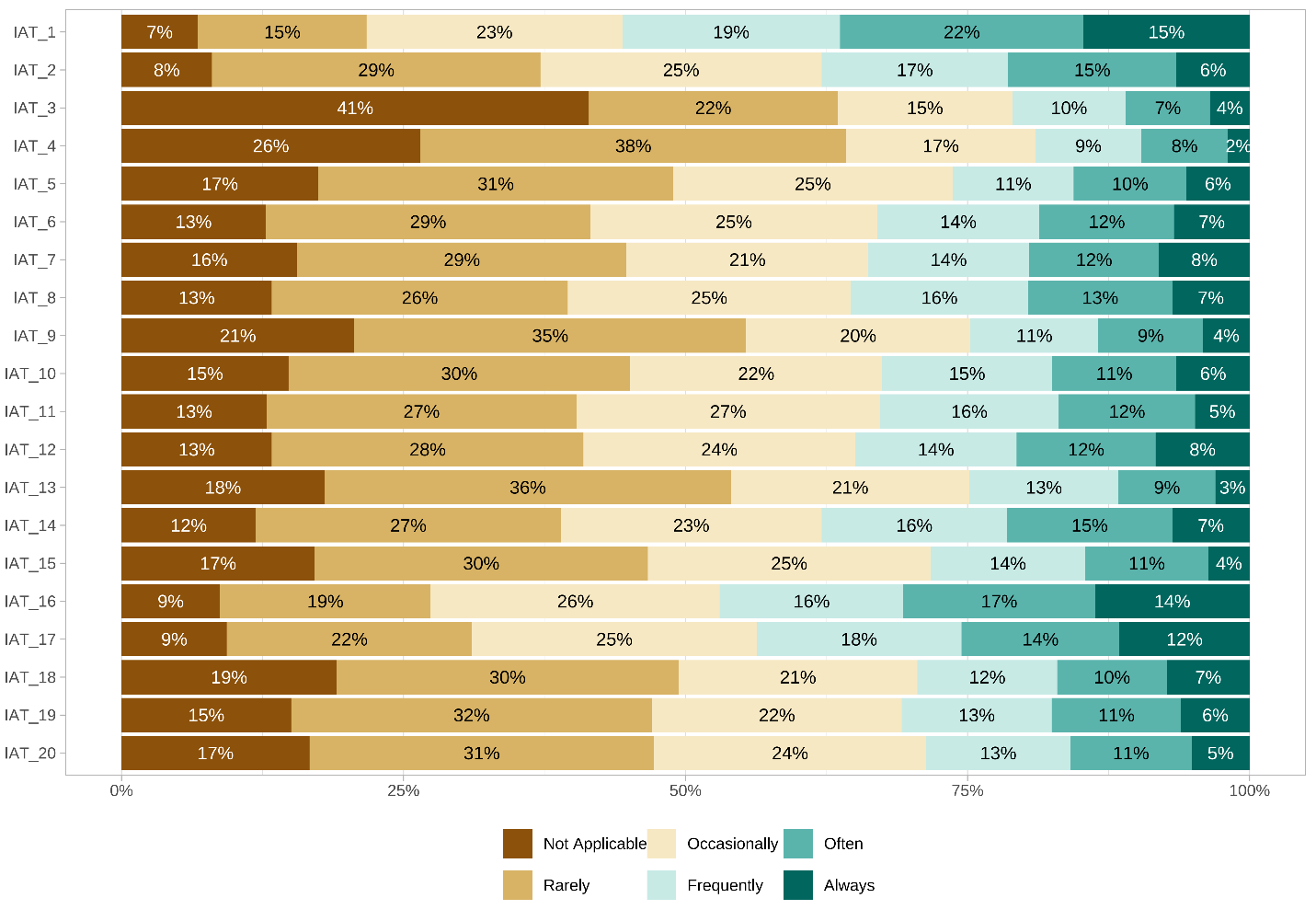


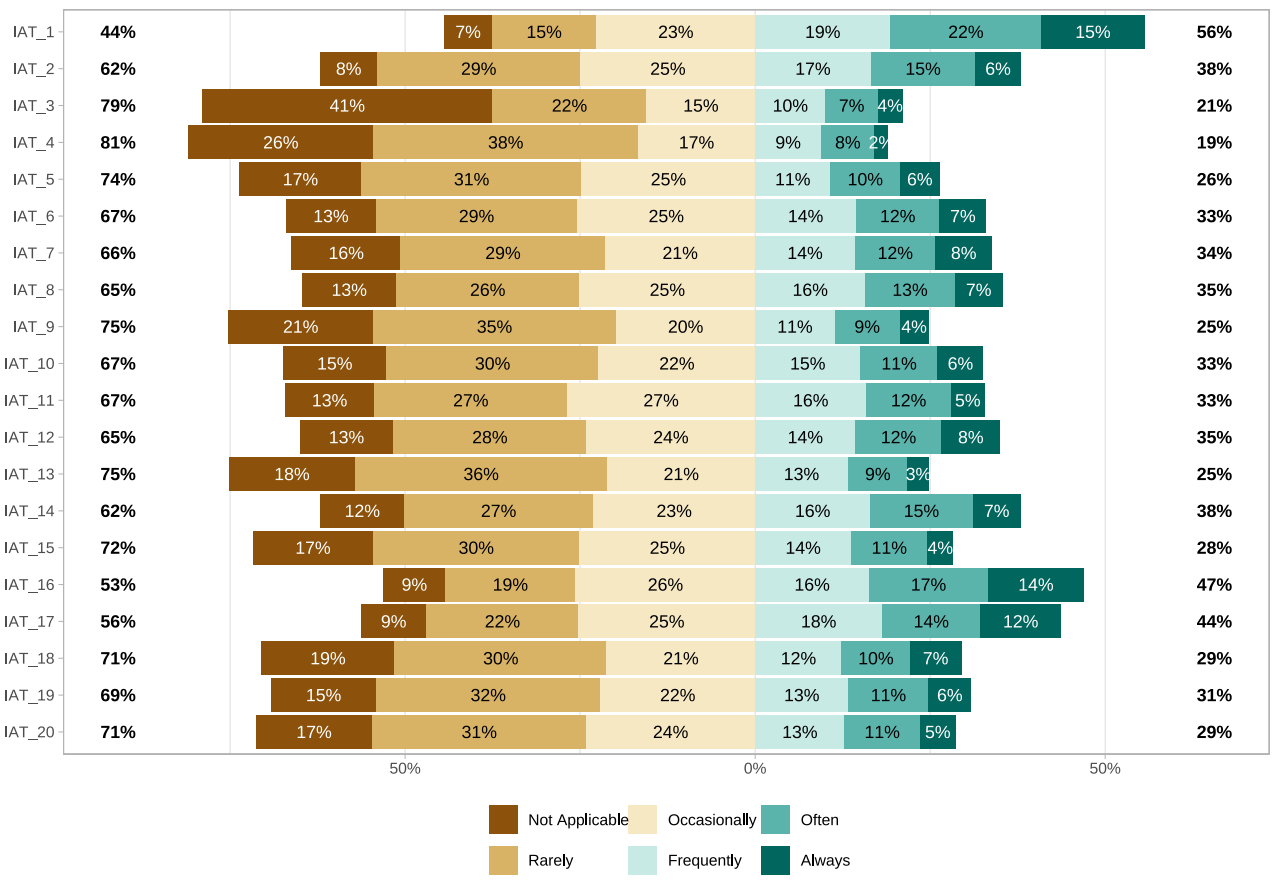


# **Figure S3:** PPCS individual items Likert plot


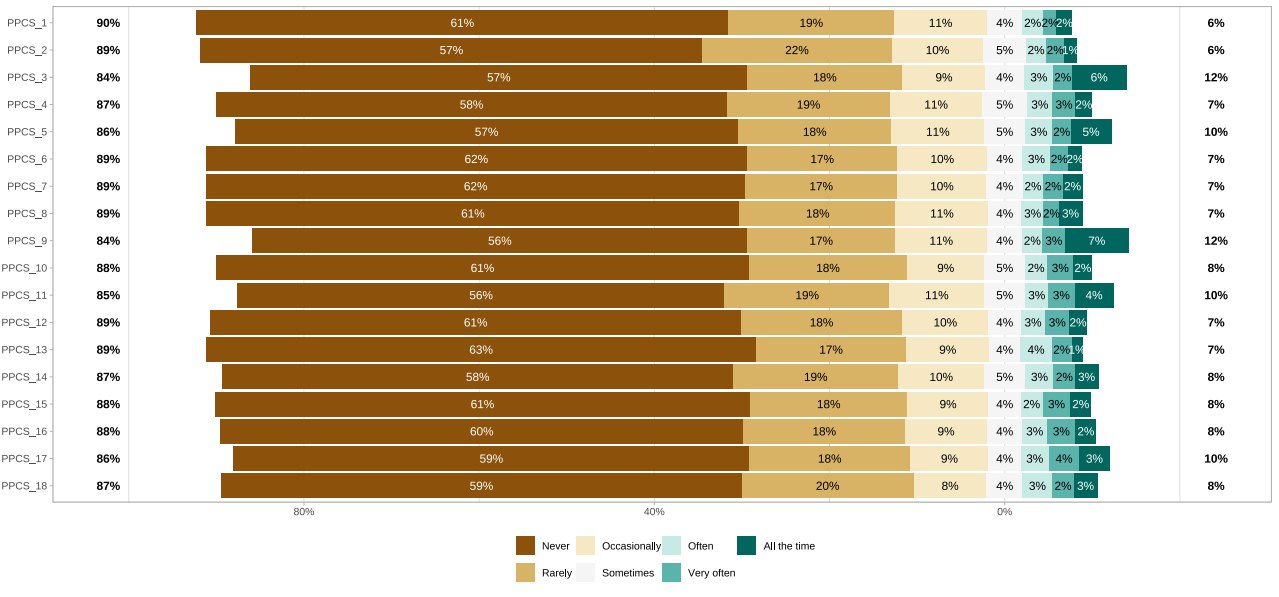

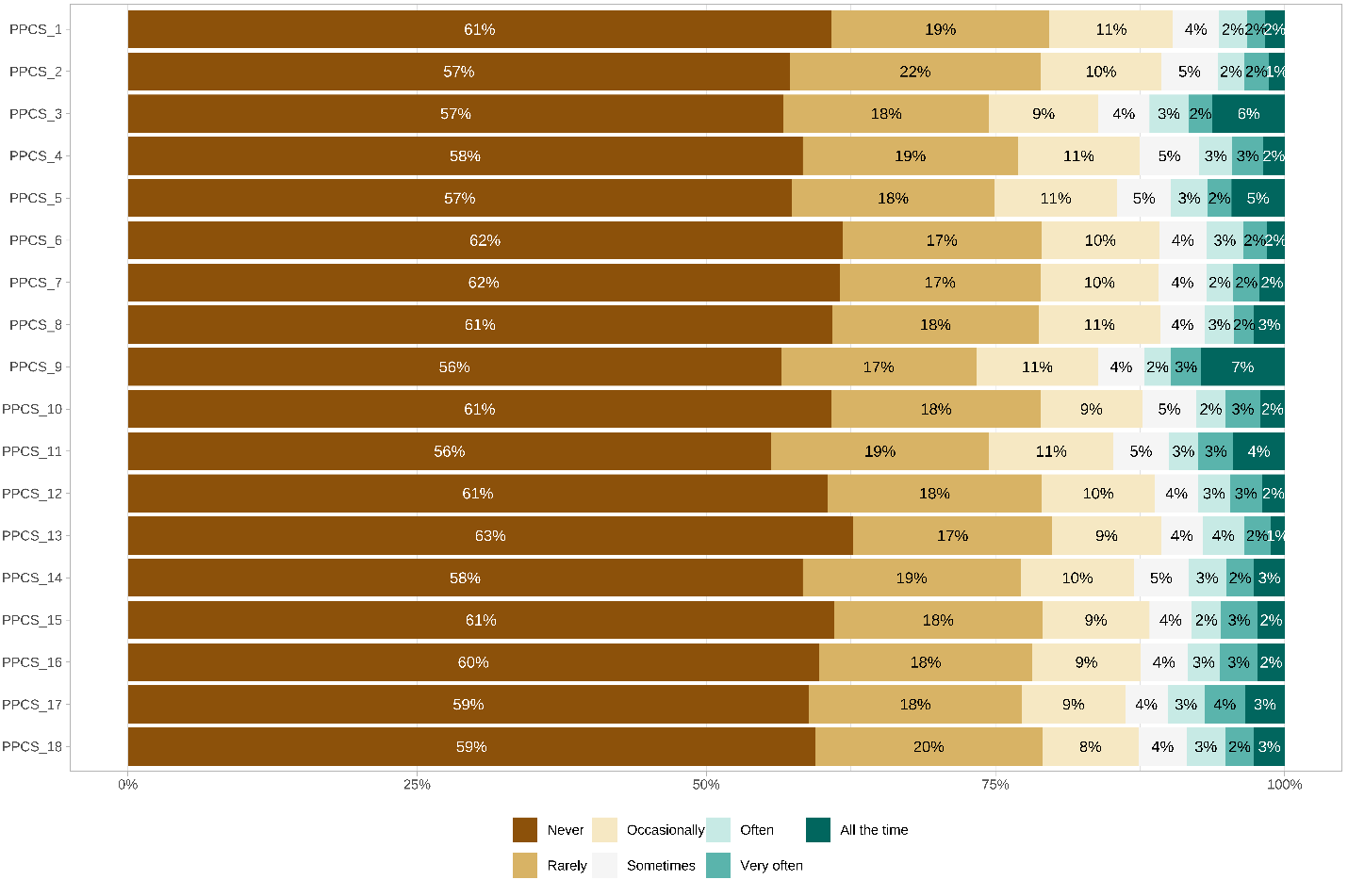


# **
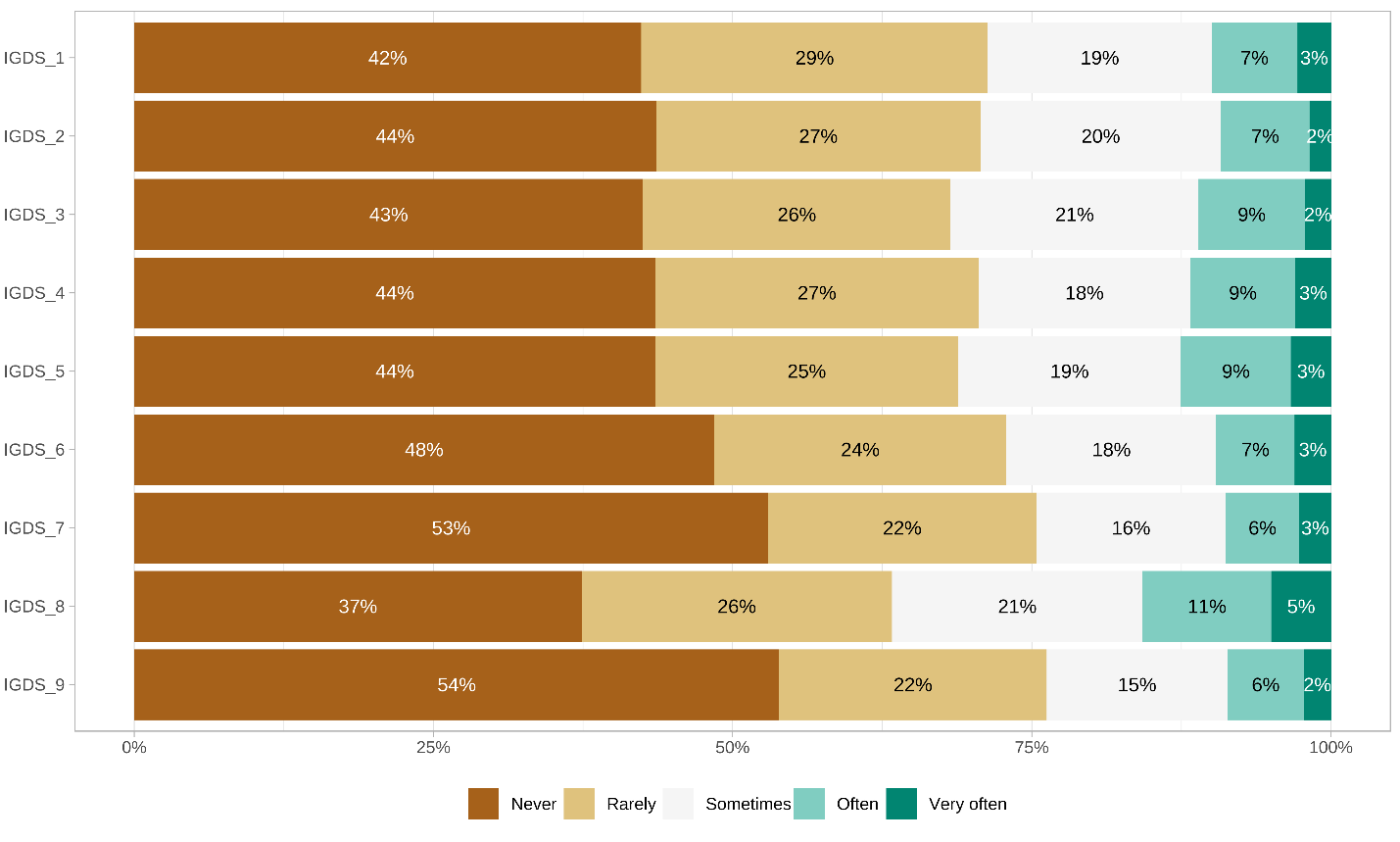
Figure S4:** IGDS individual items Likert plot


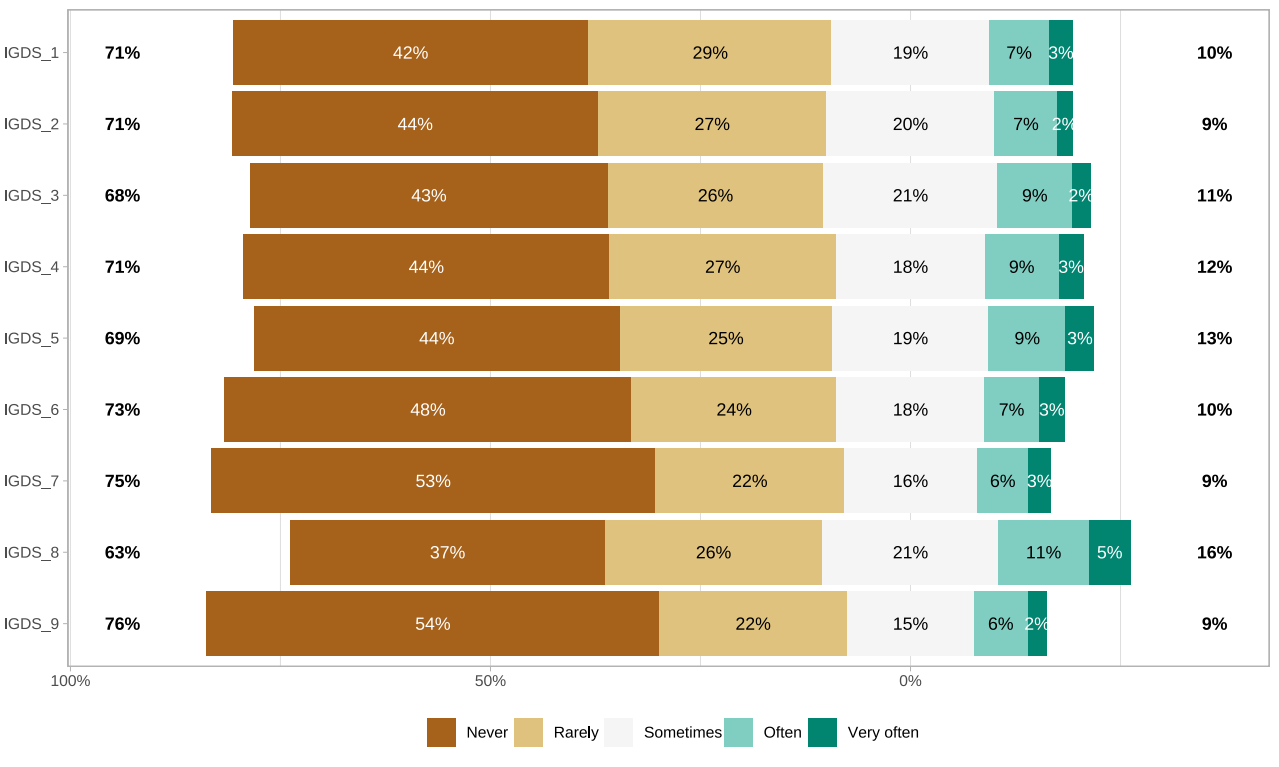


# **Figure S5**: Density plot represent the latent profile analysis


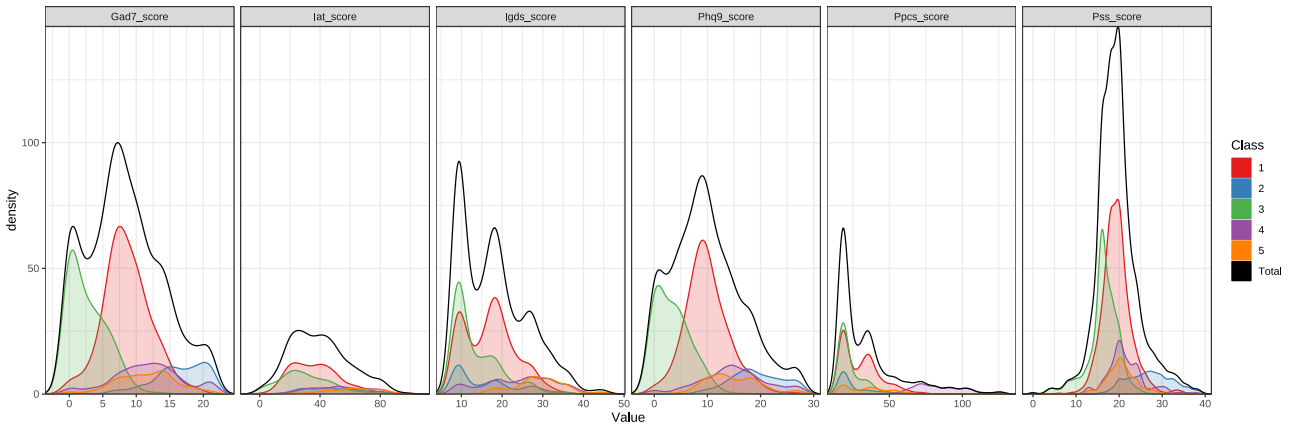

Supplement: Supplementary file 1 — Supplementary Material 1 [file 41598_2026_56057_MOESM1_ESM.docx]
